# Supplementary material for: Memory for rewards guides retrieval
Source: Commun Psychol. 2024 Apr 16;2:31. doi: 10.1038/s44271-024-00074-9 (PMC11332070; doi:10.1038/s44271-024-00074-9)
Supplement: Supplementary file 2 — Supplementary Information [file 44271_2024_74_MOESM2_ESM.pdf]

# Supplementary Material for "Memory for Rewards Guides Retrieval"

Nagel J[1,2,3], Morgan DP[1], Gürsoy, NÇ[1,2,3], Sander, S[1,2,3], Kern S[1,2,3], Feld GB[1,2,3,4]

[1] Clinical Psychology, Central Institute of Mental Health, Medical Faculty Mannheim, University of Heidelberg, Mannheim, Germany

[2] Addiction Behavior and Addiction Medicine, Central Institute of Mental Health, Medical Faculty Mannheim, University of Heidelberg, Mannheim, Germany

[3] Psychiatry and Psychotherapy, Central Institute of Mental Health, Medical Faculty Mannheim, University of Heidelberg, Mannheim, Germany

[4] Department of Psychology, University of Heidelberg, Heidelberg, Germany

* Correspondence: Email: juliane.nagel@zi-mannheim.de; gordon.feld@zi-mannheim.de

Juliane Nagel, Central Institute of Mental Health, J5, 68159 Mannheim, Germany, Tel. +49 621 1703 6164, Fax +49 621 1703 6505

Gordon Feld, Central Institute of Mental Health, J5, 68159 Mannheim, Germany, Tel. +49 621 1703 6540, Fax +49 621 1703 6505

## Supplementary Methods

*Supplementary Table 1: Inclusion criteria for all three experiments.*

| Inclusion Criteria |
|---|
| Prolific approval rating of at least 95% and participated in at least 10 studies on Prolific. |
| Country of residence, United Kingdom. |
| Aged between 18-30. |
| Education: At least A-levels or equivalent |
| Participants must use a computer or laptop to participate. |
| Participants must not have participated in the study conducted to validate the images used in this experiment, or (for experiment 2 or 3) in any of the previous experiments in this series. |

*Supplementary Table 2: Exclusion criteria for all three experiments.*

| Exclusion Criteria |
| --- |
| Participants who incorrectly answered the practice trials and/or the questions about the task in either the learning or the test phase. (For every question, they could try twice.) |
| Participants with premature button presses, reaction times below 150ms, in more than 20% of the flanker task and/or the Psychomotor Vigilance Task (PVT). (These are considered implausibly fast reaction times that indicate that participants did not participate in the task the way it was intended, e.g., random key presses.) |
| Participants who have 20% or more lapses on the PVT (i.e., reactions > 1000ms). |
| Participants who incorrectly respond on >50% of the flankers task, indicating they did not pay attention to the task. |
| Participants with a d' of 0 and below, indicating chance or worse than chance performance. |
| Participants who did not complete both parts of the experiment. |
| Participants who expected the same reward level on ≥ 50% of target trials (in experiment 1). |

*Note:* Due to a typo, 2% premature button presses instead of 20% were preregistered as exclusion criterion for the PVT/flanker task in experiment 1.

*Supplementary Table 3:* How many participants were excluded for each exclusion criterion in experiment 1. Note that several criteria can apply for the same participant, e.g., someone who did not complete the learning phase of the experiment will also not have completed the test phase of the experiment. In experiment 1, one participant was excluded because they participated twice in the study due to a coding error. One participant was excluded because they reported an education level below A levels, even though we pre-screened participants for education on Prolific.

| criterion | count |
|---|---:|
| incomplete test phase | 179 |
| incomplete study phase | 109 |
| failed validation questions twice | 30 |
| biased reward expectations | 30 |
| chance level d’ | 17 |
| >= 20% lapses PVT | 14 |
| > 20% premature responses PVT | 10 |
| education below A levels | 1 |
| participated twice | 1 |
| > 50% of flankers incorrect | 0 |
| > 20% premature responses flankers | 0 |

*Supplementary Table 4:* How many participants were excluded for each exclusion criterion in experiment 2. Note that several criteria can apply for the same participant, e.g., someone who did not complete the learning phase of the experiment will also not have completed the test phase of the experiment. In experiment 2, two participants were excluded because their *d*' was exceptionally high (*d*' = 3.41 and *d*' = 4.09, which both lie more than four standard deviations above the mean of the sample).

| | count | |
|---|---:|---:|
| criterion | congruent | incongruent |
| incomplete test phase | 148 | 108 |
| incomplete study phase | 110 | 93 |
| failed validation questions twice | 83 | 67 |
| >= 20% lapses PVT | 10 | 2 |
| > 20% premature responses PVT | 2 | 12 |
| > 50% of flankers incorrect | 1 | 0 |
| > 20% premature responses flankers | 1 | 0 |
| chance level *d*' | 1 | 2 |
| exceptionally high *d*' | 0 | 2 |

*Supplementary Table 5:* How many participants were excluded for each exclusion criterion in experiment 3. Note that several criteria can apply for the same participant, e.g., someone who did not complete the learning phase of the experiment will also not have completed the test phase of the experiment. In experiment 3, one participant was excluded because they reported an education level below A levels, even though we pre-screened participants for education on Prolific.

| criterion | count |
|---|---:|
| incomplete test phase | 187 |
| incomplete study phase | 132 |
| failed validation questions twice | 116 |
| chance level d’ | 13 |
| >= 20% lapses PVT | 4 |
| education below A levels | 2 |
| > 20% premature responses PVT | 1 |
| > 50% of flankers incorrect | 1 |
| > 20% premature responses flankers | 1 |

## Supplementary Note 1

*Supplementary Table 6:* Performance in the tasks and control measures for each of the three experiments.

| | experiment 1 | experiment 2 | | experiment 3 |
|---|---|---|---|---|
| | | congruent | incongruent | |
| **Motivated Learning Task** | | | | |
| hit rate | 0.61 (0.13) | 0.60 (0.09) | 0.60 (0.13) | 0.63 (0.13) |
| false alarm rate | 0.39 (0.13) | 0.35 (0.11) | 0.35 (0.11) | 0.33 (0.14) |
| *d*' | 0.61 (0.36) | 0.69 (0.37) | 0.66 (0.39) | 0.84 (0.57) |
| criterion | 0.00 (0.31) | 0.07 (0.22) | 0.08 (0.28) | 0.07 (0.30) |
| **PVT** | | | | |
| % lapses study | 3.13 (6.64) | 2.30 (4.52) | 1.92 (3.18) | 5.41 (9.67) |
| % prematures study | 0.34 (1.71) | 0.12 (0.72) | 0.35 (1.64) | 0.12 (0.72) |
| % lapses test | 4.04 (6.66) | 3.98 (6.80) | 5.09 (8.15) | 5.93 (8.94) |
| % prematures test | 0.18 (1.00) | 0.17 (1.71) | 0.33 (1.54) | 0.09 (0.64) |
| **Flankers** | | | | |
| % correct | 97.29 (4.69) | 98.16 (1.56) | 98.11 (1.56) | 97.79 (3.24) |
| mean rt | 519.29 (53.10) | 530.65 (52.35) | 523.94 (55.37) | 529.00 (54.79) |
| **Stanford Sleepiness Scale** | | | | |
| study | 2.12 (1.04) | 2.09 (1.01) | 2.28 (0.96) | 2.03 (0.95) |
| test | 2.09 (1.07) | 2.20 (1.13) | 2.18 (1.01) | 2.02 (1.06) |

## Supplementary Note 2

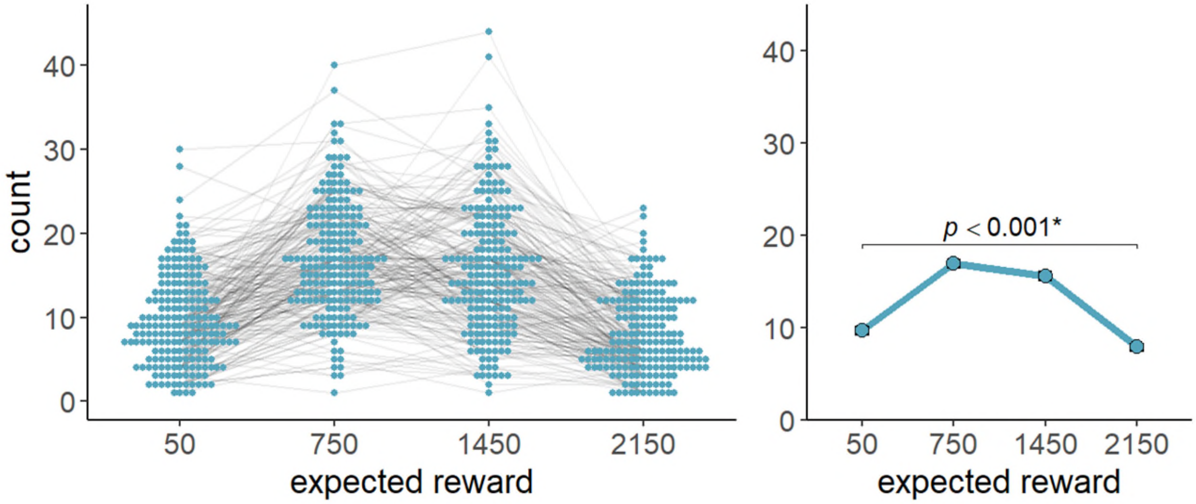


*Supplementary Figure 1:* Number of lure trials per expected reward level. The number of trials is not uniformly distributed across expected reward levels. The beeswarm plot shows the data at the participant level, with one data point per level of expected reward, and grey lines connecting dependent data points. The line plot shows group means for each level of expected reward. Black error bars show the within-subject standard error, calculated as implemented in the R package Rmisc[1,2]. The p-value for a χ^2-test for a uniform distribution is reported (see main article), with asterisks representing significance at $\alpha = .05$.

## Supplementary Note 3

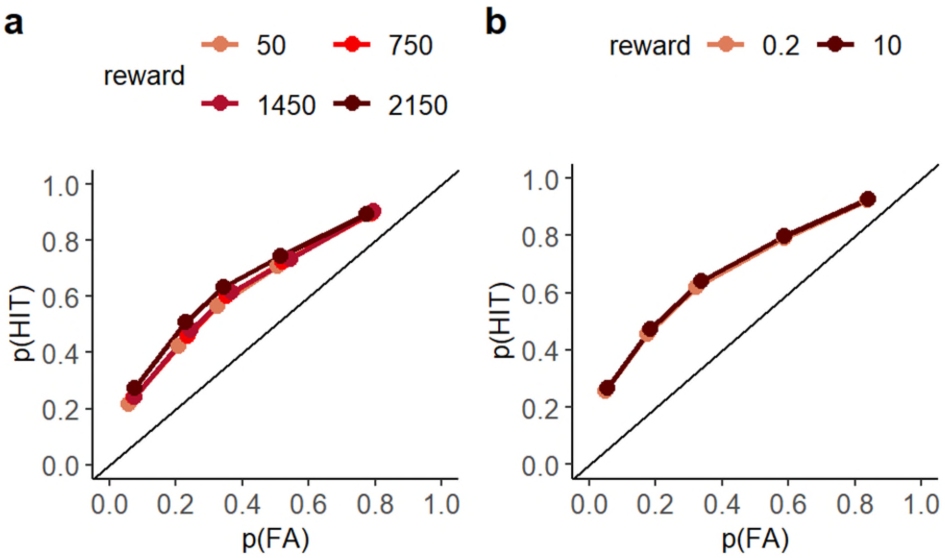


*Supplementary Figure 2:* ROC curves. **a** ROC curve for each confidence level of the different reward levels in the congruent group in experiment 2 and **b** for experiment 3. ROC curves were calculated as described in Yonelinas & Parks (2007)[3]. Decision (old/new) and confidence (guess/sure/very sure) were collapsed into a single confidence scale.

## Supplementary Note 4

When we designed experiment 1, we assumed that asking participants about the reward they expected whenever they identified a picture as old would yield a meaningful false alarm rate, that could e.g. be used to calculate measures such as $d'$. This did not work as planned, and we had to refine our methods in subsequent experiments. Because of this, our analyses for experiment 1 focus on the evaluations that informed our decisions for experiment 2 and 3. For the sake of completeness, we report the results of the preregistered analysis for experiment 1 here in the supplement. Initially, we planned to investigate the shape of the relationship between reward and memory performance (i.e., whether it is linear, quadratic or sigmoidal).

We compared the following three models using the package `brms` (version 2.20.1[4]):

- Linear: `hit rate ~ reward + (1 | participant)`, `family = gaussian()`
- Quadratic: `hit rate ~ reward + reward^2 + (1 | participant)`, `family = gaussian()`
- Sigmoidal: `hit rate ~ reward + (1 | participant)`, `family = zero_one_inflated_beta(link = "logit")`

The predictor reward was recoded as the reward divided by 1000, minus the mean reward divided by 1000. This was done to express any effects of reward in units of 1000 gems. Furthermore, we set the mean reward as zero, so that the middle reward categories would be the vertex of the quadratic function. `(1 | participant)` denotes a random per-participant intercept. All models used a default prior and ran Markov chain Monte Carlo algorithms, as implemented in package `brms`, using four Markov Chains for each model. We preregistered to use a binomial family for the model testing a sigmoidal relationship, but then realized that this kind of model does not work on the aggregated data (hit rate per participant), as it requires integers as outcome variable. Instead, we used a zero one inflated beta model, reflecting that our outcome is a probability with the limits 0 and 1. Like the binomial family, it used a logit link function.

We compared the different models by comparing their model weights using an approximation of loo (leave-one-out) cross-validation (using the function `model_weights()`

in `brms` with `weights = "loo"`). The quadratic model received the highest weight (0.937), suggesting that it describes the data best. The zero one inflated model (0.000) and the linear model (0.062) were clearly inferior to the quadratic model.

## Supplementary Note 5

For hypotheses 2 and 3 in the preregistration for experiment 2, we planned to investigate the influence of shown reward and true reward on memory strength and decision criterion, respectively. Shown reward and true reward were supposed to be entered as predictors in the same model, but we later realized that this model could not be calculated in a meaningful way. For example, while it is possible to calculate the hit rate per shown reward x true reward combination, that is not possible for the false alarms, because lures only have a shown reward level. Instead, we now report the analysis for memory strength and decision criterion with only the shown reward as predictor. For the true reward, the false alarm rate would have once again been based on the shown reward for lures, i.e., simply comparing the hit rate between shown and true reward is more meaningful.

## Supplementary Note 6

For experiment 1, we could not calculate a meaningful false alarm rate (and thus, no meaningful $d$' or criterion). However, we still report the results for $d$' and criterion in the supplement, with two alternative ways to calculate the false alarm rate: 1) The false alarm rate is calculated as the number of false alarms per expected reward level, divided by the total number of trials per expected reward level for each participant ("flexible false alarm rate"). Technically, this is not a true false alarm rate, as it pits false alarms against hits, rather than false alarms against correct rejections. 2) The false alarm rate is calculated as the number of false alarms per expected reward level, divided by a constant ("fixed false alarm rate"; 128 trials in our case).

For a detailed discussion of why we could not calculate a false alarm rate, and the issues with each of the two alternative ways to calculate a false alarm rate, see the interim discussion of experiment 1 in the main article. Note that the axes of the plots for $d$' and criterion in this supplement are on a different scale than plots for the same measures in the main article. That is because ranges of $d$' and criterion, as we calculated them here, differ from the ranges of $d$' and criterion as calculated in the main article (where calculating a meaningful false alarm rate was possible). Likewise, the axes of the plots for the decision criterion (calculated with the fixed false alarm rate) are on a different scale than the other axes of the criterion plots throughout the article.

### Flexible False Alarm Rate

The hit rate was based on the true reward, and was calculated as it was calculated for the main analyses: The number of hits divided by the number of target trials per true reward category (i.e., 32). When the hit rate or false alarm rate was 1 or 0, we corrected these values based on the assumption that each hit rate or false alarm rate was based on 32 targets/lures (according to Macmillan & Creelman (2005)[5], when the hit rate is 1, it is corrected to ($n_{\text{targets}}$ - .5) / $n_{\text{targets}}$, and when the hit rate i 0, it is corrected to .5 / $n_{\text{targets}}$).

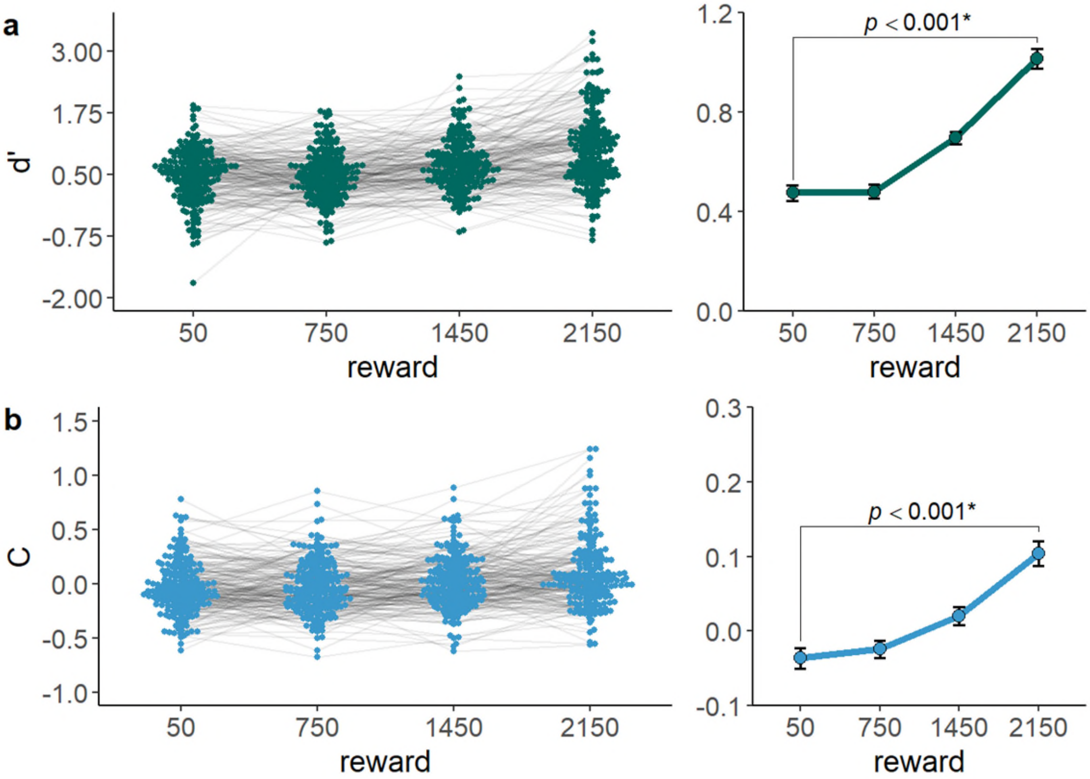


*Supplementary Figure 3:* Memory strength and criterion for experiment 1 based on a flexible false alarm rate. **a** Memory strength increased with increasing reward. **b** The higher the reward, the more lenient the decision criterion was. In the beeswarm plots of each panel, colored dots represent data per participant, with light grey lines connecting the dependent data points. In the line plots of each panel, colored dots show group means. Black error bars show the within-subject standard error, calculated as implemented in the R package Rmisc[1,2]. P-values for the reward effect in separate models for memory strength and decision criterion are reported (see Supplementary Tables 7 and 8). Asterisks represent significance at $\alpha = .05$.

*Supplementary Table 7:* Full model output for the following linear mixed model: `d' ~ reward + (reward | participant)`. d' was calculated based on a flexible false alarm rate.

| effect | group | term | estimate | standard error | statistic | df | p-value |
|---|---|---|---|---|---|---|---|
| fixed | | Intercept | 0.38 | 0.04 | 10.74 | 199.00 | p < .001 |
| fixed | | reward | 0.26 | 0.02 | 10.80 | 199.00 | p < .001 |
| random | participant | sd Intercept | 0.36 | | | | |
| random | participant | cor Intercept.reward | -0.15 | | | | |
| random | participant | sd reward | 0.23 | | | | |
| random | Residual | sd Observation | 0.40 | | | | |

*Supplementary Table 8:* Full model output for the following linear mixed model: `C ~ reward + (reward | participant)`. Criterion was calculated based on a flexible false alarm rate.

| effect | group | term | estimate | standard error | statistic | df | p-value |
|---|---|---|---|---|---|---|---|
| fixed | | Intercept | -0.06 | 0.02 | -3.51 | 199.00 | p < .001 |
| fixed | | reward | 0.07 | 0.01 | 6.28 | 199.00 | p < .001 |
| random | participant | sd Intercept | 0.18 | | | | |
| random | participant | cor Intercept.reward | -0.22 | | | | |
| random | participant | sd reward | 0.10 | | | | |
| random | Residual | sd Observation | 0.17 | | | | |

Using a flexible false alarm rate, we analyzed the effect of reward on the resulting $d'$ with the following linear mixed model: `d' ~ reward + (reward | participant)`. As the reward increased, $d'$ increased, $\beta = 0.26$, $SE = 0.02$, $t(199.00) = 10.80$, $p < .001$ (see Supplementary Figure 3a and Supplementary Table 7). That is, memory sensitivity for the highest reward category ($M = 1.02$, $SD = 0.76$) was significantly higher than for the lowest

reward category ($M = 0.47, SD = 0.54$), $t(199) = 10.27, p < .001, d_z = 0.73$, 95% $CI$ [0.57, 0.88]. We analyzed the effect of reward on criterion calculated based on a flexible false alarm rate with the following linear mixed model: `C ~ reward + (reward | participant)`. As the reward increased, participants were more likely to identify a picture as old, $\beta = 0.07$, $SE = 0.01$, $t(199.00) = 6.28$, $p < .001$ (see Supplementary Figure 3b and Supplementary Table 8). That is, for the highest reward category ($M = 0.10$, $SD = 0.32$) criterion was significantly lower (more lenient) than for the lowest reward category ($M = -0.04, SD = 0.24$), $t(199) = 6.02, p < .001, d_z = 0.43$, 95% $CI$ [0.28, 0.57].

## Fixed False Alarm Rate

To have a common reference frame for the false alarm rate and the hit rate, for this analysis, we calculated the hit rate (based on the true reward) as the number of hits divided by a constant (128 trials, i.e., all target trials). When the hit rate or false alarm rate was 1 or 0, we corrected these values based on the assumption that each hit rate or false alarm rate was based on 128 targets/lures.

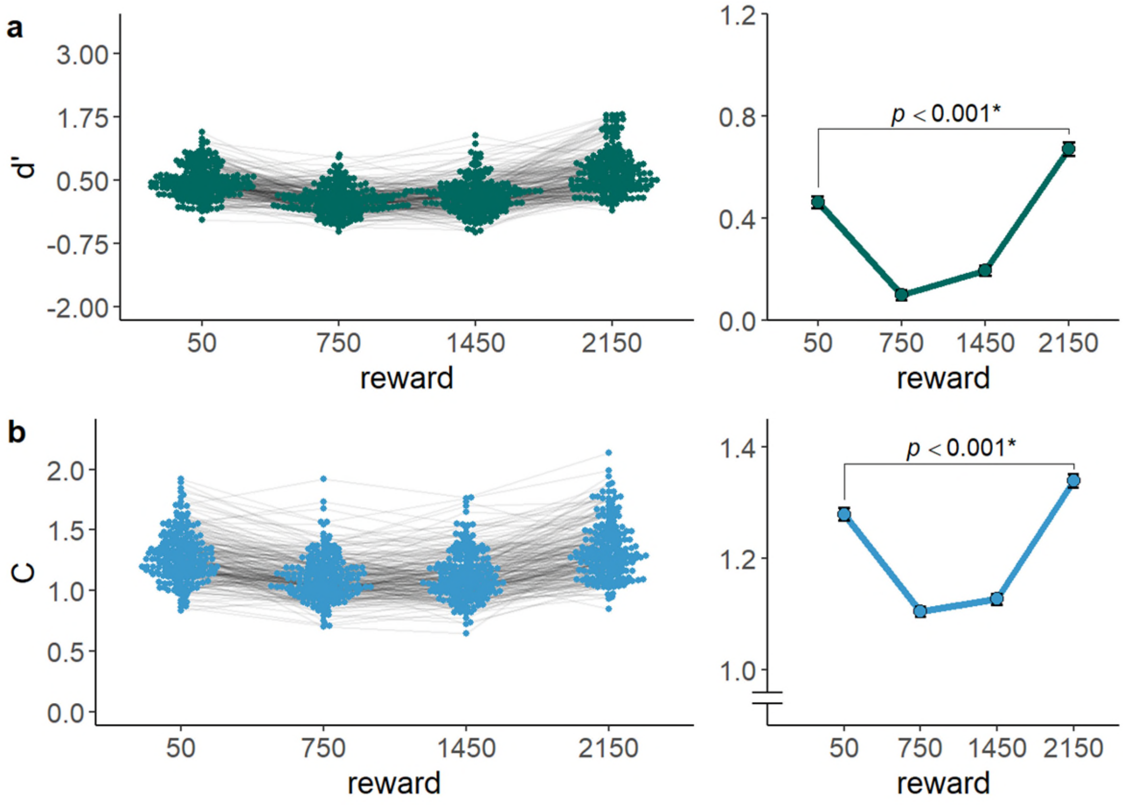


*Supplementary Figure 4:* Memory strength and criterion for experiment 1 based on a fixed

false alarm rate. **a** Memory strength increased with increasing reward. **b** The higher the reward, the more lenient the decision criterion was. In the beeswarm plots of each panel, colored dots represent data per participant, with light grey lines connecting the dependent data points. In the line plots of each panel, colored dots show group means. Black error bars show the within-subject standard error, calculated as implemented in the R package Rmisc[1,2]. P-values for the (quadratic) reward effect in separate models for memory strength and decision criterion per group are reported (see Supplementary Tables 9 and 10). Asterisks represent significance at $\alpha = .05$.

*Supplementary Table 9:* Full model output for the following linear mixed model: *`d' ~ reward + reward^2 (1 | participant)`*. d' was calculated based on a fixed false alarm rate.

| effect | group | term | estimate | standard error | statistic | df | p-value |
|---|---|---|---|---|---|---|---|
| fixed | | Intercept | 0.50 | 0.02 | 22.95 | 517.33 | p < .001 |
| fixed | | reward | -0.84 | 0.05 | -18.53 | 399.01 | p < .001 |
| fixed | | reward^2 | 0.43 | 0.02 | 20.87 | 510.45 | p < .001 |
| random | participant | sd Intercept | 0.10 | | | | |
| random | participant | cor Intercept.reward^2 | -0.33 | | | | |
| random | participant | sd reward^2 | 0.08 | | | | |
| random | Residual | sd Observation | 0.27 | | | | |

*Supplementary Table 10:* Full model output for the following linear mixed model: *`C ~ reward + reward^2 (reward^2 | participant)`*. Criterion C was calculated based on a fixed false alarm rate.

| effect | group | term | estimate | standard error | statistic | df | p-value |
|---|---|---|---|---|---|---|---|
| fixed | | Intercept | 1.30 | 0.01 | 89.78 | 333.91 | p < .001 |
| fixed | | reward | -0.41 | 0.02 | -18.82 | 399.00 | p < .001 |
| fixed | | reward^2 | 0.20 | 0.01 | 20.25 | 510.49 | p < .001 |
| random | participant | sd Intercept | 0.15 | | | | |
| random | participant | cor Intercept.reward^2 | -0.26 | | | | |
| random | participant | sd reward^2 | 0.04 | | | | |
| random | Residual | sd Observation | 0.13 | | | | |

Using a fixed false alarm rate, we analyzed the effect of reward on the resulting $d'$ with the following linear mixed model: `d' ~ reward + (1 | participant)`. This model still resulted in a singular fit, and a visual inspection of the data indicated that a quadratic model would provide a better fit. So instead, we fit the following linear mixed model: `d' ~ reward + reward^2 + (reward^2 | participant)`. As the reward increased, $d'$ increased, i.e., both the linear $\beta = -0.84$, $SE = 0.05$, $t(399.01) = -18.53$, $p < .001$ and quadratic reward predictor $\beta = 0.43$, $SE = 0.02$, $t(510.45) = 20.87$, $p < .001$ had a significant effect (see Supplementary Figure 4a and Supplementary Table 9). That is, memory sensitivity for the highest reward category ($M = 0.67$, $SD = 0.42$) was significantly higher than for the lowest reward category ($M = 0.46$, $SD = 0.32$), $t(199) = 5.74$, $p < .001$, $d_z = 0.41$, 95% $CI$ [0.26, 0.55]. We analyzed the effect of reward on criterion calculated based on a fixed false alarm rate with the following linear mixed model: `C ~ reward + reward^2 (reward^2 | participant)`. As the reward increased, participants were more likely to identify a picture as old, i.e., both the linear $\beta = -0.41$, $SE = 0.02$, $t(399.00) = -18.82$, $p < .001$ and quadratic reward predictor $\beta = 0.20$, $SE = 0.01$, $t(510.49) = 20.25$, $p < .001$ had a significant effect (see Supplemenary Figure 4b and Supplementary Table 10). That is, for

the highest reward category ($M = 1.34$, $SD = 0.23$) criterion was significantly lower (more lenient) than for the lowest reward category ($M = 1.28$, $SD = 0.21$), $t(199) = 3.49$, $p <$ .001, $d_z = 0.25$, 95% $CI$ [0.11, 0.39].

### Conclusion

Despite not being able to calculate a meaningful false alarm rate based on participants' reward expectations (see interim discussion experiment 1 in the main article), we report analyses of memory strength and criterion for experiment 1 for interested readers. We use two different alternatives to calculate the false alarm rate: a flexible false alarm rate, and a fixed false alarm rate (see description above). Both alternatives have limitations (see interim discussion experiment 1 in the main article), which is why the memory strength and criterion results for experiment 1 need to be interpreted with caution. For both versions of the false alarm rate, we find that memory strength and criterion increase as the reward increases. The relationship between reward and memory strength/decision criterion is linear when using a flexible false alarm rate, and follows a U-shape when using a fixed false alarm rate (even though memory strength/decision criterion are still significantly different when comparing the lowest and highest reward level). However, for the fixed false alarm rate, this most likely reflects the fact that participants were strongly biased towards choosing the medium reward categories (see Supplementary Figure 1). This results in an increased false alarm rate that is based on the fact that much more trials fall into the medium reward categories. These results further highlight the difficulty of using reward expectations as a basis for a reward-related false alarm rate.

## Supplementary Note 7

Here, we report the full output of the mixed models reported in the main manuscript. Note that the full output of the alternative drift diffusion analysis can be found in Supplementary Note 9.

### Experiment 1

*Supplementary Table 11:* Full model output for the following generalized linear mixed model (logit link function): `hit (0 or 1) ~ reward + (reward | participant) + (1 | image)`, run on target trials only.

| effect | group | term | estimate | standard error | statistic | p-value |
|---|---|---|---|---|---|---|
| fixed | | Intercept | 0.43 | 0.05 | 8.55 | p < .001 |
| fixed | | reward | 0.11 | 0.02 | 5.59 | p < .001 |
| random | image | sd Intercept | 0.65 | | | |
| random | participant | sd Intercept | 0.55 | | | |
| random | participant | cor Intercept.reward | 0.19 | | | |
| random | participant | sd reward | 0.10 | | | |

*Supplementary Table 12:* Full model output for the following generalized linear mixed model (logit link function): `hit (0 or 1) ~ reward * confidence + (reward + confidence || participant) + (confidence | image)`, run on target trials only.

| effect | group | term | estimate | standard error | statistic | p-value |
|---|---|---|---|---|---|---|
| fixed | | Intercept | 0.19 | 0.06 | 3.35 | p < .001 |
| fixed | | reward | 0.05 | 0.03 | 1.67 | p = .096 |
| fixed | | confidence | 0.45 | 0.07 | 6.36 | p < .001 |
| fixed | | reward:confidence | 0.06 | 0.02 | 2.70 | p = .007 |
| random | image | sd Intercept | 0.50 | | | |
| random | image | cor Intercept.confidence | 0.81 | NA | | |
| random | image | sd confidence | 0.23 | | | |
| random | participant | sd confidence | 0.85 | | | |
| random | participant | sd reward | 0.10 | | | |
| random | participant | sd Intercept | 0.54 | | | |

*Supplementary Table 13:* Full model output for the following generalized linear mixed model (logit link function): `hit (0 or 1) ~ reward + (1 | participant) + (1 | image)`, run on target trials with confidence level "guess" only.

| effect | group | term | estimate | standard error | statistic | p-value |
|---|---|---|---|---|---|---|
| fixed | | Intercept | 0.19 | 0.06 | 3.38 | p < .001 |
| fixed | | reward | 0.05 | 0.03 | 1.77 | p = .076 |
| random | image | sd Intercept | 0.44 | | | |
| random | participant | sd Intercept | 0.48 | | | |

*Supplementary Table 14:* Full model output for the following generalized linear mixed model (logit link function): `hit (0 or 1) ~ reward + (1 | participant) + (1 | image)`, run on target trials with confidence level "sure" only.

| effect | group | term | estimate | standard error | statistic | p-value |
|---|---|---|---|---|---|---|
| fixed | | Intercept | 0.63 | 0.08 | 8.39 | p < .001 |
| fixed | | reward | 0.09 | 0.03 | 2.85 | p = .004 |
| random | image | sd Intercept | 0.76 | | | |
| random | participant | sd Intercept | 0.77 | | | |

*Supplementary Table 15:* Full model output for the following generalized linear mixed model (logit link function): `hit (0 or 1) ~ reward + (1 | participant) + (1 | image)`, run on target trials with confidence level "very sure" only.

| effect | group | term | estimate | standard error | statistic | p-value |
|---|---|---|---|---|---|---|
| fixed | | Intercept | 1.12 | 0.13 | 8.46 | p < .001 |
| fixed | | reward | 0.19 | 0.04 | 4.86 | p < .001 |
| random | image | sd Intercept | 0.88 | | | |
| random | participant | sd Intercept | 1.59 | | | |

*Supplementary Table 16:* Full model output for the following generalized linear mixed model (logit link function): `false alarm (0 or 1) ~ expected reward * confidence + (expected reward || participant) + (1 | image)`, run on trials where participants responded "old".

| effect | group | term | estimate | standard error | statistic | p-value |
|---|---|---|---|---|---|---|
| fixed | | Intercept | 0.01 | 0.04 | 0.29 | p = .775 |
| fixed | | expected reward | 0.01 | 0.03 | 0.31 | p = .757 |
| fixed | | confidence | -0.49 | 0.04 | -13.81 | p < .001 |
| fixed | | expected reward:confidence | -0.10 | 0.03 | -3.73 | p < .001 |
| random | image | sd Intercept | 0.16 | | | |
| random | participant | sd expected reward | 0.14 | | | |
| random | participant | sd Intercept | 0.24 | | | |

*Supplementary Table 17:* Full model output for the following generalized linear mixed model (logit link function): `false alarm (0 or 1) ~ expected reward + (1 | participant)`, run on trials where participants responded "old" with confidence level "guess" only.

| effect | group | term | estimate | standard error | statistic | p-value |
|---|---|---|---|---|---|---|
| fixed | | Intercept | -0.02 | 0.03 | -0.45 | p = .651 |
| fixed | | expected reward | -0.05 | 0.03 | -1.70 | p = .089 |
| random | participant | sd Intercept | 0.07 | | | |

*Supplementary Table 18:* Full model output for the following generalized linear mixed model (logit link function): `false alarm (0 or 1) ~ expected reward + (1 | participant)`, run on trials where participants responded "old" with confidence level "sure" only.

| effect | group | term | estimate | standard error | statistic | p-value |
|---|---|---|---|---|---|---|
| fixed | | Intercept | -0.38 | 0.05 | -7.74 | p < .001 |
| fixed | | expected reward | -0.01 | 0.03 | -0.31 | p = .753 |
| random | participant | sd Intercept | 0.29 | | | |

*Supplementary Table 19:* Full model output for the following generalized linear mixed model (logit link function): `false alarm (0 or 1) ~ expected reward + (1 | participant)`, run on trials where participants responded "old" with confidence level "very sure" only.

| effect | group | term | estimate | standard error | statistic | p-value |
|---|---|---|---|---|---|---|
| fixed | | Intercept | -1.25 | 0.09 | -14.15 | p < .001 |
| fixed | | expected reward | -0.15 | 0.04 | -3.27 | p = .001 |
| random | participant | sd Intercept | 0.68 | | | |

*Supplementary Table 20:* Full model output for the following linear mixed model: `expected reward ~ reward * confidence + (confidence * reward || participant)`, run on hit trials only.

| effect | group | term | estimate | standard error | statistic | df | p-value |
|---|---|---|---|---|---|---|---|
| fixed | | Intercept | 867.74 | 17.91 | 48.46 | 649.74 | p < .001 |
| fixed | | reward | -8.01 | 11.36 | -0.71 | 1108.19 | p = .480 |
| fixed | | confidence | 240.64 | 13.60 | 17.70 | 703.98 | p < .001 |
| fixed | | reward:confidence | 45.37 | 8.64 | 5.25 | 1249.44 | p < .001 |
| random | participant | sd Intercept | 141.01 | | | | |
| random | participant | sd confidence | 97.57 | | | | |
| random | participant | sd reward | 46.46 | | | | |
| random | participant | sd confidence:reward | 31.92 | | | | |
| random | Residual | sd Observation | 641.17 | | | | |

*Supplementary Table 21:* Full model output for the following linear mixed model: `expected reward ~ reward + (reward || participant)`, run on hit trials with confidence level “guess” only.

| effect | group | term | estimate | standard error | statistic | df | p-value |
|---|---|---|---|---|---|---|---|
| fixed | | Intercept | 876.48 | 21.16 | 41.42 | 461.73 | p < .001 |
| fixed | | reward | 5.44 | 12.39 | 0.44 | 380.55 | p = .661 |
| random | participant | sd Intercept | 175.20 | | | | |
| random | participant | sd reward | 29.25 | | | | |
| random | Residual | sd Observation | 651.64 | | | | |

*Supplementary Table 22:* Full model output for the following linear mixed model: `expected reward ~ reward + (reward || participant)`, run on hit trials with confidence level "sure" only.

| effect | group | term | estimate | standard error | statistic | df | p-value |
|---|---|---|---|---|---|---|---|
| fixed | | Intercept | 1096.24 | 16.55 | 66.25 | 577.64 | p < .001 |
| fixed | | reward | 16.07 | 11.01 | 1.46 | 492.57 | p = .145 |
| random | participant | sd Intercept | 112.17 | | | | |
| random | participant | sd reward | 35.60 | | | | |
| random | Residual | sd Observation | 614.39 | | | | |

*Supplementary Table 23:* Full model output for the following linear mixed model: `expected reward ~ reward + (reward || participant)`, run on hit trials with confidence level "very sure" only.

| effect | group | term | estimate | standard error | statistic | df | p-value |
|---|---|---|---|---|---|---|---|
| fixed | | Intercept | 1350.85 | 21.54 | 62.70 | 393.43 | p < .001 |
| fixed | | reward | 90.97 | 12.32 | 7.39 | 340.10 | p < .001 |
| random | participant | sd Intercept | 191.15 | | | | |
| random | participant | sd reward | 61.27 | | | | |
| random | Residual | sd Observation | 652.50 | | | | |

*Supplementary Table 24:* Full model output for the following linear mixed model: *`expected reward ~ confidence + (confidence || participant)`*, run on false alarm trials only.

| effect | group | term | estimate | standard error | statistic | df | p-value |
|---|---|---|---|---|---|---|---|
| fixed | | Intercept | 850.93 | 14.64 | 58.11 | 235.39 | p < .001 |
| fixed | | confidence | 272.60 | 12.70 | 21.46 | 207.23 | p < .001 |
| random | participant | sd Intercept | 158.49 | | | | |
| random | participant | sd confidence | 108.31 | | | | |
| random | Residual | sd Observation | 629.48 | | | | |

*Supplementary Table 25:* Full model output for the following generalized linear mixed model (logit link function): *`hit (0 or 1) ~ duration + (duration || participant) + (1 | image)`*, run on target trials only.

| effect | group | term | estimate | standard error | statistic | p-value |
|---|---|---|---|---|---|---|
| fixed | | Intercept | 0.18 | 0.09 | 2.11 | p = .035 |
| fixed | | duration | 0.18 | 0.04 | 4.72 | p < .001 |
| random | image | sd Intercept | 0.65 | | | |
| random | participant | sd duration | 0.15 | | | |
| random | participant | sd Intercept | 0.49 | | | |

## Experiment 2

*Supplementary Table 26:* Full model output for the following generalized linear mixed model (logit link function): `hit (0 or 1) ~ reward + (reward | participant) + (reward | image)`, run on target trials in the congruent group only.

| effect | group | term | estimate | standard error | statistic | p-value |
|---|---|---|---|---|---|---|
| fixed | | Intercept | 0.32 | 0.06 | 5.69 | p < .001 |
| fixed | | reward | 0.13 | 0.03 | 4.94 | p < .001 |
| random | image | sd Intercept | 0.70 | | | |
| random | image | cor Intercept.reward | -0.76 | | | |
| random | image | sd reward | 0.13 | | | |
| random | participant | sd Intercept | 0.40 | | | |
| random | participant | cor Intercept.reward | -0.37 | | | |
| random | participant | sd reward | 0.10 | | | |

*Supplementary Table 27:* Full model output for the following generalized linear mixed model (logit link function): *`hit (0 or 1) ~ reward * experiment + (reward + experiment || participant) + (1 | image)`*, run on target trials of experiment 1 and experiment 2 (congruent group) only.

| effect | group | term | estimate | standard error | statistic | p-value |
|---|---|---|---|---|---|---|
| fixed | | Intercept | 0.37 | 0.04 | 8.79 | p < .001 |
| fixed | | reward | 0.13 | 0.02 | 7.88 | p < .001 |
| fixed | | experiment | -0.11 | 0.07 | -1.53 | p = .125 |
| fixed | | reward:experiment | 0.04 | 0.03 | 1.33 | p = .182 |
| random | image | sd Intercept | 0.62 | | | |
| random | participant | sd Intercept | 0.50 | | | |
| random | participant | cor Intercept.reward | 0.04 | | | |
| random | participant | sd reward | 0.10 | | | |

*Supplementary Table 28:* Full model output for the following generalized linear mixed model (logit link function): *`hit (0 or 1) ~ true reward + shown reward + (shown reward | participant) + (1 | image)`*, run on target trials for the incongruent group only.

| effect | group | term | estimate | standard error | statistic | p-value |
|---|---|---|---|---|---|---|
| fixed | | Intercept | 0.25 | 0.08 | 3.27 | p = .001 |
| fixed | | true reward | 0.02 | 0.02 | 0.96 | p = .337 |
| fixed | | shown reward | 0.15 | 0.04 | 4.05 | p < .001 |
| random | image | sd Intercept | 0.59 | | | |
| random | participant | sd Intercept | 0.62 | | | |
| random | participant | cor Intercept.shown reward | -0.39 | | | |
| random | participant | sd shown reward | 0.29 | | | |

*Supplementary Table 29:* Full model output for the following generalized linear mixed model (logit link function): *hit (0 or 1) ~ shown reward * group + (shown reward + group | participant) + (1 | image*), run on target trials only.

| effect | group | term | estimate | standard error | statistic | p-value |
|---|---|---|---|---|---|---|
| fixed | | Intercept | 0.29 | 0.04 | 6.58 | p < .001 |
| fixed | | shown reward | 0.15 | 0.02 | 7.09 | p < .001 |
| fixed | | group | 0.03 | 0.08 | 0.36 | p = .717 |
| fixed | | shown reward:group | -0.01 | 0.04 | -0.13 | p = .895 |
| random | image | sd Intercept | 0.59 | | | |
| random | participant | sd group | 0.66 | | | |
| random | participant | sd shown reward | 0.17 | | | |
| random | participant | sd Intercept | 0.33 | | | |

*Supplementary Table 30:* Full model output for the following generalized linear mixed model (logit link function): *`false alarm (0 or 1) ~ reward * group + (reward | participant) + (reward || image)`*, run on lure trials only.

| effect | group | term | estimate | standard error | statistic | p-value |
|---|---|---|---|---|---|---|
| fixed | | Intercept | -0.95 | 0.06 | -15.86 | p < .001 |
| fixed | | shown reward | 0.13 | 0.03 | 4.92 | p < .001 |
| fixed | | group | 0.14 | 0.10 | 1.43 | p = .153 |
| fixed | | shown reward:group | -0.14 | 0.05 | -2.76 | p = .006 |
| random | image | sd shown reward | 0.13 | | | |
| random | image | sd Intercept | 1.02 | | | |
| random | participant | sd Intercept | 0.59 | | | |
| random | participant | cor Intercept.shown reward | -0.18 | | | |
| random | participant | sd shown reward | 0.22 | | | |

*Supplementary Table 31:* Full model output for the following generalized linear mixed model (logit link function): *`false alarm (0 or 1) ~ reward + (reward | participant) + (1 | image)`*, run on lure trials in the congruent group only.

| effect | group | term | estimate | standard error | statistic | p-value |
|---|---|---|---|---|---|---|
| fixed | | Intercept | -0.90 | 0.08 | -11.50 | p < .001 |
| fixed | | shown reward | 0.07 | 0.03 | 2.15 | p = .031 |
| random | image | sd Intercept | 1.07 | | | |
| random | participant | sd Intercept | 0.57 | | | |
| random | participant | cor Intercept.shown reward | -0.08 | | | |
| random | participant | sd shown reward | 0.04 | | | |

*Supplementary Table 32:* Full model output for the following generalized linear mixed model (logit link function): *false alarm (0 or 1) ~ reward + (reward | participant) + (1 | image)*, run on lure trials in the incongruent group only.

| effect | group | term | estimate | standard error | statistic | p-value |
|---|---|---|---|---|---|---|
| fixed | | Intercept | -1.01 | 0.08 | -12.87 | p < .001 |
| fixed | | shown reward | 0.20 | 0.04 | 4.61 | p < .001 |
| random | image | sd Intercept | 0.97 | | | |
| random | participant | sd Intercept | 0.59 | | | |
| random | participant | cor Intercept.shown reward | -0.29 | | | |
| random | participant | sd shown reward | 0.31 | | | |

*Supplementary Table 33:* Full model output for the following linear mixed model: *d' ~ reward + (reward || participant)*, run on the congruent group only.

| effect | group | term | estimate | standard error | statistic | df | p-value |
|---|---|---|---|---|---|---|---|
| fixed | | Intercept | 0.64 | 0.04 | 15.14 | 140.67 | p < .001 |
| fixed | | reward | 0.06 | 0.02 | 2.54 | 140.67 | p = .012 |
| random | participant | sd Intercept | 0.33 | | | | |
| random | participant | sd reward | 0.09 | | | | |
| random | Residual | sd Observation | 0.32 | | | | |

*Supplementary Table 34:* Full model output for the following linear mixed model: *`d' ~ reward + (1 | participant)`*, run on the congruent group only, where reward is treated as a categorical predictor.

| effect | group | term | estimate | standard error | statistic | df | p-value |
|---|---|---|---|---|---|---|---|
| fixed | | Intercept | 0.66 | 0.05 | 14.02 | 223.81 | p < .001 |
| fixed | | reward 750 | 0.02 | 0.05 | 0.43 | 306.00 | p = .669 |
| fixed | | reward 1450 | 0.02 | 0.05 | 0.34 | 306.00 | p = .738 |
| fixed | | reward 2150 | 0.13 | 0.05 | 2.90 | 306.00 | p = .004 |
| random | participant | sd Intercept | 0.35 | | | | |
| random | Residual | sd Observation | 0.33 | | | | |

*Supplementary Table 35:* Full model output for the following linear mixed model: *`d' ~ shown reward + (shown reward | participant)`*, run on the incongruent group only.

| effect | group | term | estimate | standard error | statistic | df | p-value |
|---|---|---|---|---|---|---|---|
| fixed | | Intercept | 0.73 | 0.05 | 14.99 | 101.00 | p < .001 |
| fixed | | shown reward | -0.04 | 0.02 | -1.61 | 101.00 | p = .110 |
| random | participant | sd Intercept | 0.38 | | | | |
| random | participant | cor Intercept.shown reward | -0.31 | | | | |
| random | participant | sd shown reward | 0.11 | | | | |
| random | Residual | sd Observation | 0.35 | | | | |

*Supplementary Table 36:* Full model output for the following linear mixed model: *`criterion ~ reward + (reward | participant)`*, run on the congruent group only.

| effect | group | term | estimate | standard error | statistic | df | p-value |
|---|---|---|---|---|---|---|---|
| fixed | | Intercept | 0.14 | 0.03 | 5.12 | 102.00 | p < .001 |
| fixed | | reward | -0.06 | 0.01 | -4.84 | 101.99 | p < .001 |
| random | participant | sd Intercept | 0.24 | | | | |
| random | participant | cor Intercept.reward | -0.57 | | | | |
| random | participant | sd reward | 0.06 | | | | |
| random | Residual | sd Observation | 0.16 | | | | |

*Supplementary Table 37:* Full model output for the following linear mixed model: *`criterion ~ shown reward + (shown reward | participant)`*, run on the incongruent group only.

| effect | group | term | estimate | standard error | statistic | df | p-value |
|---|---|---|---|---|---|---|---|
| fixed | | Intercept | 0.20 | 0.03 | 5.99 | 101.00 | p < .001 |
| fixed | | shown reward | -0.11 | 0.02 | -5.19 | 101.00 | p < .001 |
| random | participant | sd Intercept | 0.29 | | | | |
| random | participant | cor Intercept.shown reward | -0.37 | | | | |
| random | participant | sd shown reward | 0.17 | | | | |
| random | Residual | sd Observation | 0.19 | | | | |

*Supplementary Table 38:* Full model output for the following generalized linear mixed model (logit link function): *hit (0 or 1) ~ reward * confidence + (reward + confidence | participant) + (confidence | image)*, run on target trials for the congruent group only.

| effect | group | term | estimate | standard error | statistic | p-value |
|---|---|---|---|---|---|---|
| fixed | | Intercept | -0.14 | 0.10 | -1.39 | p = .166 |
| fixed | | true reward | 0.08 | 0.04 | 1.69 | p = .090 |
| fixed | | confidence | 0.55 | 0.10 | 5.66 | p < .001 |
| fixed | | true reward:confidence | 0.05 | 0.04 | 1.32 | p = .187 |
| random | image | sd Intercept | 0.49 | | | |
| random | image | cor Intercept.confidence | 0.64 | | | |
| random | image | sd confidence | 0.19 | | | |
| random | participant | sd Intercept | 0.80 | | | |
| random | participant | cor Intercept.true reward | 0.31 | | | |
| random | participant | cor Intercept.confidence | -0.77 | | | |
| random | participant | sd true reward | 0.10 | | | |
| random | participant | cor true reward.confidence | -0.52 | | | |
| random | participant | sd confidence | 0.82 | | | |

*Supplementary Table 39:* Full model output for the following generalized linear mixed model (logit link function): *hit (0 or 1) ~ true reward * confidence + (confidence || participant) + (1 | image)*, run on target trials for the incongruent group only.

| effect | group | term | estimate | standard error | statistic | p-value |
|---|---|---|---|---|---|---|
| fixed | | Intercept | -0.09 | 0.09 | -1.00 | p = .317 |
| fixed | | true reward | 0.05 | 0.04 | 1.22 | p = .221 |
| fixed | | confidence | 0.58 | 0.09 | 6.53 | p < .001 |
| fixed | | true reward:confidence | -0.02 | 0.03 | -0.57 | p = .572 |
| random | image | sd Intercept | 0.60 | | | |
| random | participant | sd confidence | 0.73 | | | |
| random | participant | sd Intercept | 0.69 | | | |

*Supplementary Table 40:* Full model output for the following generalized linear mixed model (logit link function): `hit (0 or 1) ~ shown reward * confidence + (shown reward + confidence | participant) + (confidence || image)`, run on target trials for the incongruent group only.

| effect | group | term | estimate | standard error | statistic | p-value |
|---|---|---|---|---|---|---|
| fixed | | Intercept | -0.24 | 0.11 | -2.09 | p = .037 |
| fixed | | shown reward | 0.19 | 0.05 | 3.69 | p < .001 |
| fixed | | confidence | 0.61 | 0.10 | 5.99 | p < .001 |
| fixed | | shown reward:confidence | -0.05 | 0.04 | -1.37 | p = .170 |
| random | image | sd confidence | 0.17 | | | |
| random | image | sd Intercept | 0.58 | | | |
| random | participant | sd Intercept | 0.94 | | | |
| random | participant | cor Intercept.shown reward | -0.23 | | | |
| random | participant | cor Intercept.confidence | -0.71 | | | |
| random | participant | sd shown reward | 0.28 | | | |
| random | participant | cor shown reward.confidence | -0.02 | | | |
| random | participant | sd confidence | 0.87 | | | |

*Supplementary Table 41:* Full model output for the following generalized linear mixed model (logit link function): *`false alarm (0 or 1) ~ shown reward * confidence + (shown reward | participant) + (1 | image)`*, run on lure trials for the congruent group only.

| effect | group | term | estimate | standard error | statistic | p-value |
|---|---|---|---|---|---|---|
| fixed | | Intercept | -0.61 | 0.09 | -6.66 | $p < .001$ |
| fixed | | shown reward | 0.05 | 0.05 | 1.04 | $p = .300$ |
| fixed | | confidence | -0.31 | 0.05 | -5.75 | $p < .001$ |
| fixed | | shown reward:confidence | 0.03 | 0.04 | 0.71 | $p = .480$ |
| random | image | sd Intercept | 1.03 | | | |
| random | participant | sd Intercept | 0.57 | | | |
| random | participant | cor Intercept.shown reward | -0.12 | | | |
| random | participant | sd shown reward | 0.07 | | | |

*Supplementary Table 42:* Full model output for the following generalized linear mixed model (logit link function): *false alarm (0 or 1) ~ shown reward * confidence + (shown reward + confidence | participant) + (1 | image)*, run on lure trials for the incongruent group only.

| effect | group | term | estimate | standard error | statistic | p-value |
|---|---|---|---|---|---|---|
| fixed | | Intercept | -0.49 | 0.12 | -4.08 | p < .001 |
| fixed | | shown reward | 0.20 | 0.06 | 3.51 | p < .001 |
| fixed | | confidence | -0.53 | 0.10 | -5.52 | p < .001 |
| fixed | | shown reward:confidence | 0.01 | 0.04 | 0.26 | p = .794 |
| random | image | sd Intercept | 0.91 | | | |
| random | participant | sd Intercept | 0.94 | | | |
| random | participant | cor Intercept.shown reward | -0.39 | | | |
| random | participant | cor Intercept.confidence | -0.65 | | | |
| random | participant | sd shown reward | 0.31 | | | |
| random | participant | cor shown reward.confidence | 0.29 | | | |
| random | participant | sd confidence | 0.75 | | | |

*Supplementary Table 43:* Full model output for the following linear mixed model: `confidence ~ group * image type + (image type | participant)`, run on all trials in experiment 2.

| effect | group | term | estimate | standard error | statistic | df | p-value |
|---|---|---|---|---|---|---|---|
| fixed | | Intercept | 1.02 | 0.03 | 39.68 | 203.00 | p < .001 |
| fixed | | group | 0.05 | 0.05 | 0.91 | 203.00 | p = .365 |
| fixed | | image type | 0.10 | 0.01 | 9.37 | 203.01 | p < .001 |
| fixed | | group:image type | -0.01 | 0.02 | -0.66 | 203.01 | p = .510 |
| random | participant | sd Intercept | 0.36 | | | | |
| random | participant | cor Intercept.image type | -0.28 | | | | |
| random | participant | sd image type | 0.13 | | | | |
| random | Residual | sd Observation | 0.67 | | | | |

*Supplementary Table 44:* Full model output for the following generalized linear mixed model (logit link function): `hit (0 or 1) ~ duration + (duration | participant) + (duration || image)`, run on target trials only in the congruent group.

| effect | group | term | estimate | standard error | statistic | p-value |
|---|---|---|---|---|---|---|
| fixed | | Intercept | 0.19 | 0.12 | 1.65 | p = .100 |
| fixed | | duration | 0.14 | 0.05 | 2.50 | p = .012 |
| random | image | sd duration | 0.06 | | | |
| random | image | sd Intercept | 0.59 | | | |
| random | participant | sd Intercept | 0.52 | | | |
| random | participant | cor Intercept.duration | -0.70 | | | |
| random | participant | sd duration | 0.19 | | | |

*Supplementary Table 45:* Full model output for the following generalized linear mixed model (logit link function): `hit (0 or 1) ~ duration + (duration | participant) + (duration || image)`, run on target trials only in the incongruent group.

| effect | group | term | estimate | standard error | statistic | p-value |
|---|---|---|---|---|---|---|
| fixed | | Intercept | 0.15 | 0.12 | 1.27 | p = .203 |
| fixed | | duration | 0.15 | 0.06 | 2.56 | p = .010 |
| random | image | sd duration | 0.11 | | | |
| random | image | sd Intercept | 0.54 | | | |
| random | participant | sd Intercept | 0.54 | | | |
| random | participant | cor Intercept.duration | -0.34 | | | |
| random | participant | sd duration | 0.23 | | | |

## Experiment 3

*Supplementary Table 46:* Full model output for the following generalized linear mixed model (logit link function): *hit (0 or 1) ~ confidence * reward + (confidence + reward | participant) + (confidence || image)*, run on target trials only.

| effect | group | term | estimate | standard error | statistic | p-value |
|---|---|---|---|---|---|---|
| fixed | | Intercept | -0.11 | 0.06 | -2.00 | p = .046 |
| fixed | | confidence | 0.92 | 0.07 | 13.12 | p < .001 |
| fixed | | reward | 0.11 | 0.05 | 2.43 | p = .015 |
| fixed | | confidence:reward | -0.01 | 0.04 | -0.24 | p = .810 |
| random | image | sd confidence | 0.17 | | | |
| random | image | sd Intercept | 0.48 | | | |
| random | participant | sd Intercept | 0.64 | | | |
| random | participant | cor Intercept.confidence | -0.57 | | | |
| random | participant | cor Intercept.reward | 0.04 | | | |
| random | participant | sd confidence | 0.89 | | | |
| random | participant | cor confidence.reward | 0.08 | | | |
| random | participant | sd reward | 0.16 | | | |

*Supplementary Table 47:* Full model output for the following generalized linear mixed model (logit link function): *false alarm (0 or 1) ~ confidence * reward + (confidence + reward || participant) + (confidence || image)*, run on lure trials only.

| effect | group | term | estimate | standard error | statistic | p-value |
|---|---|---|---|---|---|---|
| fixed | | Intercept | -0.60 | 0.07 | -8.31 | p < .001 |
| fixed | | confidence | -0.39 | 0.08 | -4.84 | p < .001 |
| fixed | | reward | 0.10 | 0.05 | 2.25 | p = .024 |
| fixed | | confidence:reward | -0.03 | 0.05 | -0.63 | p = .527 |
| random | image | sd confidence | 0.55 | | | |
| random | image | sd Intercept | 0.70 | | | |
| random | participant | sd reward | 0.16 | | | |
| random | participant | sd confidence | 0.96 | | | |
| random | participant | sd Intercept | 0.83 | | | |

## Supplementary Note 8

Here, we report exploratory analyses, where duration is added as an additional predictor to the models supporting our main analyses.

### Experiment 1

With exposure duration added to the model (Supplementary Table 48; see Supplementary Table 11 for comparison), the results do not change: Hits still increase as the reward increases. Hits also increase as the exposure duration increases, but there is no interaction between reward and duration.

*Supplementary Table 48:* Full model output for the following generalized linear mixed model (logit link function): `hit (0 or 1) ~ reward * duration + (reward | participant) + (1 | image)`, run on target trials only.

| effect | group | term | estimate | standard error | statistic | p-value |
|---|---|---|---|---|---|---|
| fixed | | Intercept | -0.08 | 0.14 | -0.61 | p = .543 |
| fixed | | reward | 0.26 | 0.10 | 2.60 | p = .009 |
| fixed | | duration | 0.26 | 0.06 | 3.99 | p < .001 |
| fixed | | reward:duration | -0.08 | 0.05 | -1.55 | p = .120 |
| random | image | sd Intercept | 0.65 | | | |
| random | participant | sd Intercept | 0.55 | | | |
| random | participant | cor Intercept.reward | 0.18 | | | |
| random | participant | sd reward | 0.10 | | | |

### Experiment 2

With duration added as a predictor for the effect of reward on hits in the congruent group, the model had to be reduced further due to convergence issues. This means that it was not possible to include a slope for the highest level interaction. In contrast to the model without duration as a predictor (Supplementary Table 49; see Supplementary Table 26 for

comparison), there is no effect of reward on hits anymore. Hits increase as duration increases, but there is no reward x duration interaction.

*Supplementary Table 49:* Full model output for the following generalized linear mixed model (logit link function): `hit (0 or 1) ~ reward * duration + (reward || participant) + (1 | image)`, run on target trials only.

| effect | group | term | estimate | standard error | statistic | p-value |
|---|---|---|---|---|---|---|
| fixed | | Intercept | -0.04 | 0.18 | -0.23 | p = .822 |
| fixed | | true reward | 0.22 | 0.13 | 1.62 | p = .105 |
| fixed | | duration | 0.18 | 0.09 | 2.01 | p = .044 |
| fixed | | true reward:duration | -0.04 | 0.07 | -0.56 | p = .573 |
| random | image | sd Intercept | 0.60 | | | |
| random | participant | sd true reward | 0.07 | | | |
| random | participant | sd Intercept | 0.37 | | | |

## Experiment 3

While analyses for experiment 3 rely on t-tests, when adding duration as a predictor, we need a linear mixed model. When investigating the joint effect of reward and duration on hits in experiment 3, while hits increase as duration increases, this does not change the main finding that hits increase as the reward increases.

*Supplementary Table 50:* Full model output for the following generalized linear mixed model (logit link function): *hit (0 or 1) ~ reward * duration + (reward + duration | participant) + (1 | image)*, run on target trials only.

| effect | group | term | estimate | standard error | statistic | p-value |
|---|---|---|---|---|---|---|
| fixed | | Intercept | 0.60 | 0.05 | 12.14 | p < .001 |
| fixed | | reward | 0.10 | 0.03 | 3.21 | p = .001 |
| fixed | | duration | -0.05 | 0.03 | -1.75 | p = .081 |
| fixed | | reward:duration | 0.09 | 0.06 | 1.62 | p = .104 |
| random | image | sd Intercept | 0.47 | | | |
| random | participant | sd Intercept | 0.60 | | | |
| random | participant | cor Intercept.reward | -0.05 | | | |
| random | participant | cor Intercept.duration | -0.96 | | | |
| random | participant | sd reward | 0.13 | | | |
| random | participant | cor reward.duration | -0.22 | | | |
| random | participant | sd duration | 0.03 | | | |

## Supplementary Note 9

Here, we report detailed model results and additional plots accompanying the alternative drift diffusion model analysis.

### Experiment 1

*Supplementary Table 51:* Full model output for the following linear mixed model: `alpha ~ expected reward + (expected reward | participant)`, run on the parameter estimates of a drift diffusion model per participant, where boundary separation $\alpha$, the starting point $\beta$, the drift rate $\delta$, and the non-decision time $\tau$ varied by expected reward level. The model was run on all trials where participants said a picture was 'old' (i.e., where they expected to receive a reward) in experiment 1.

| effect | group | term | estimate | standard error | statistic | df | p-value |
|---|---|---|---|---|---|---|---|
| fixed | | Intercept | 2.06 | 0.03 | 66.88 | 160.00 | p < .001 |
| fixed | | reward | -0.02 | 0.03 | -0.72 | 160.00 | p < .472 |
| random | participant | sd Intercept | 0.23 | | | | |
| random | participant | cor Intercept.reward | -0.34 | | | | |
| random | participant | sd reward | 0.29 | | | | |
| random | Residual | sd Observation | 0.36 | | | | |

*Supplementary Table 52:* Full model output for the following linear mixed model: `beta ~ expected reward + (expected reward | participant)`, run on the parameter estimates of a drift diffusion model per participant, where boundary separation α, the starting point β, the drift rate δ, and the non-decision time τ varied by expected reward level. The model was run on all trials where participants said a picture was 'old' (i.e., where they expected to receive a reward) in experiment 1.

| effect | group | term | estimate | standard error | statistic | df | p-value |
|---|---|---|---|---|---|---|---|
| fixed | | Intercept | 0.52 | 0.01 | 73.61 | 160.00 | p < .001 |
| fixed | | reward | 0.00 | 0.01 | -0.65 | 160.00 | p = .513 |
| random | participant | sd Intercept | 0.05 | | | | |
| random | participant | cor Intercept.reward | -0.89 | | | | |
| random | participant | sd reward | 0.05 | | | | |
| random | Residual | sd Observation | 0.08 | | | | |

*Supplementary Table 53:* Full model output for the following linear mixed model: `delta ~ expected reward + (expected reward | participant)`, run on the parameter estimates of a drift diffusion model per participant, where boundary separation $\alpha$, the starting point $\beta$, the drift rate $\delta$, and the non-decision time $\tau$ varied by expected reward level. The model was run on all trials where participants said a picture was 'old' (i.e., where they expected to receive a reward) in experiment 1.

| effect | group | term | estimate | standard error | statistic | df | p-value |
|---|---|---|---|---|---|---|---|
| fixed | | Intercept | 0.06 | 0.02 | 2.87 | 160.00 | p = .005 |
| fixed | | reward | 0.17 | 0.02 | 8.27 | 160.00 | p < .001 |
| random | participant | sd Intercept | 0.11 | | | | |
| random | participant | cor Intercept.reward | -0.79 | | | | |
| random | participant | sd reward | 0.17 | | | | |
| random | Residual | sd Observation | 0.29 | | | | |

*Supplementary Table 54:* Full model output for the following linear mixed model: `tau ~ expected reward + (expected reward | participant)`, run on the parameter estimates of a drift diffusion model per participant, where boundary separation $\alpha$, the starting point $\beta$, the drift rate $\delta$, and the non-decision time $\tau$ varied by expected reward level. The model was run on all trials where participants said a picture was 'old' (i.e., where they expected to receive a reward) in experiment 1.

| effect | group | term | estimate | standard error | statistic | df | p-value |
|---|---|---|---|---|---|---|---|
| fixed | | Intercept | 1.05 | 0.02 | 51.40 | 160.00 | p < .001 |
| fixed | | reward | -0.02 | 0.01 | -1.88 | 160.00 | p = .062 |
| random | participant | sd Intercept | 0.24 | | | | |
| random | participant | cor Intercept.reward | -0.60 | | | | |
| random | participant | sd reward | 0.06 | | | | |
| random | Residual | sd Observation | 0.13 | | | | |

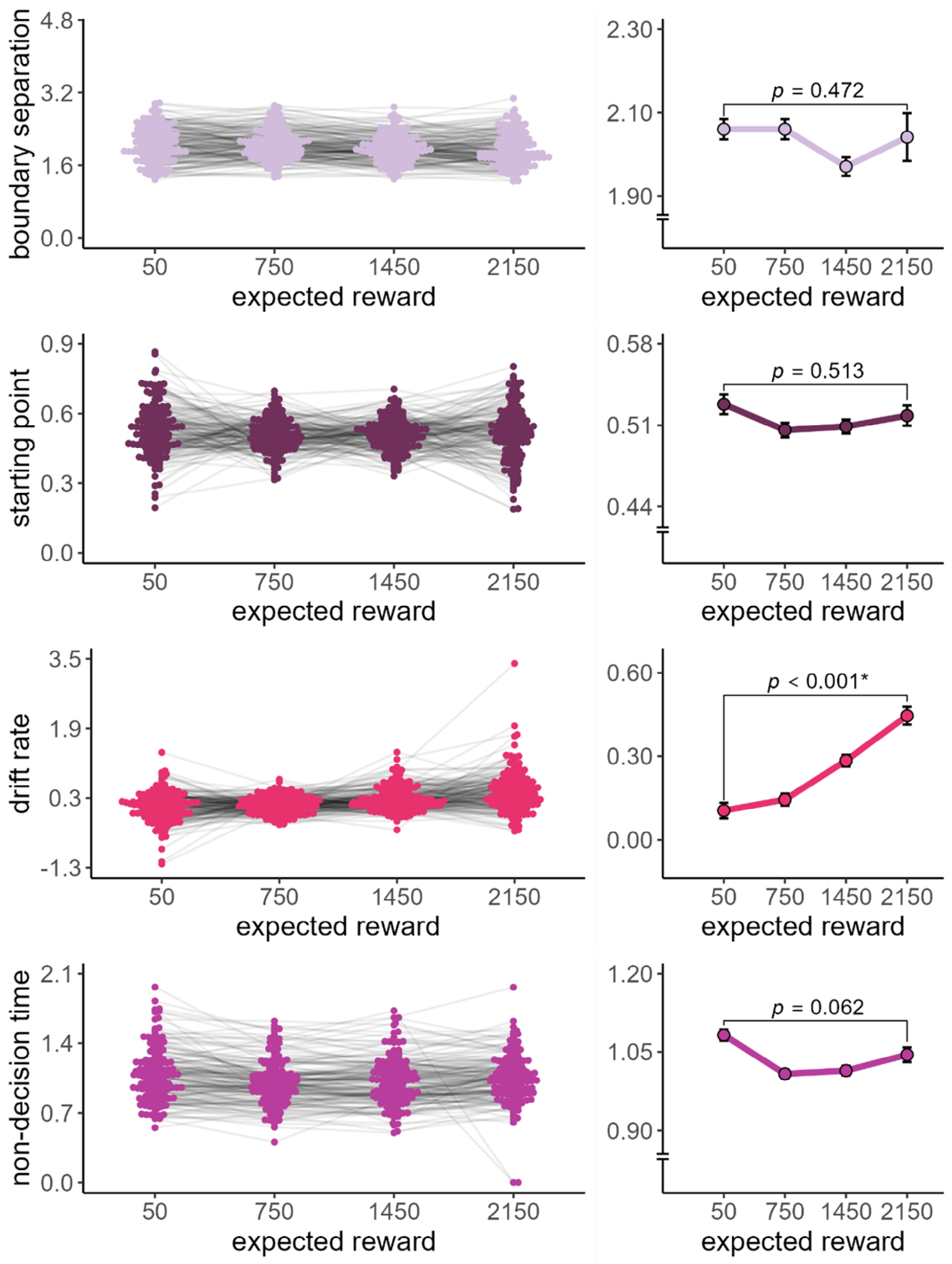


*Supplementary Figure 5:* Parameter estimates for each expected reward level in experiment 1. Estimates are based on a drift diffusion model run on $n$ = 161 participants with the outcome 'hit' (upper boundary) or 'false alarm' (lower boundary), where boundary separation $\alpha$, the starting point $\beta$, the drift rate $\delta$, and the non-decision time $\tau$ varied by expected reward level. **a** $\alpha$ parameter estimates. There was no statistically

significant effect of reward on boundary separation. In the beeswarm plot, light purple dots represent the $\alpha$ estimate per expected reward level for each participant. Light grey lines connect the dots belonging to the same participant. The line plot shows the mean $\alpha$ estimate for each expected reward level. The p-value for the expected reward effect in the mixed model from Supplementary Table 51 is reported. Note that for this data visualization, two outliers for the highest reward level are not shown in the individual plot, because they would have distorted the y-axis. The $\alpha$ values of the two outliers are 6.33 and 11.32. **b** $\beta$ parameter estimates. There was no significant effect of reward on starting point. In the beeswarm plot, dark purple dots represent the $\beta$ estimate per expected reward level for each participant. Light grey lines connect the dots belonging to the same participant. The line plot shows the mean $\beta$ estimate for each expected reward level. The p-value for the expected reward effect in the mixed model from Supplementary Table 52 is reported. **c** $\delta$ parameter estimates. The drift rate increased as the expected reward increased. In the beeswarm plot, pink dots represent the $\delta$ estimate per expected reward level for each participant. Light grey lines connect the dots belonging to the same participant. The line plot shows the mean $\delta$ estimate for each reward level. The p-value for the expected reward effect in the mixed model from Supplementary Table 53 is reported. **d** $\tau$ parameter estimates. Descriptively, the non-decision time decreased as the expected reward increased. In the beeswarm plot, purple dots represent the $\tau$ estimate per expected reward level for each participant. Light grey lines connect the dots belonging to the same participant. The line plot shows the mean $\tau$ estimate for each reward level. The p-value for the expected reward effect in the mixed model from Supplementary Table 54 is reported. Black error bars show the within-subject standard error, calculated as implemented in the R package Rmisc[60,61]. Asterisks represent significance at $\alpha = .05$.

## Experiment 2

### *Congruent*

*Supplementary Table 55:* Full model output for the following linear mixed model: `alpha ~ reward + (reward | participant)`, run on the parameter estimates of a drift diffusion model per participant in the congruent condition of experiment 2, where boundary separation $\alpha$, the starting point $\beta$, the drift rate $\delta$, and the non-decision time $\tau$ varied by reward level.

| effect | group | term | estimate | standard error | statistic | df | p-value |
|---|---|---|---|---|---|---|---|
| fixed | | Intercept | 2.04 | 0.03 | 61.40 | 102.00 | p < .001 |
| fixed | | reward | 0.00 | 0.01 | 0.45 | 102.00 | p = .656 |
| random | participant | sd Intercept | 0.32 | | | | |
| random | participant | cor Intercept.reward | -0.14 | | | | |
| random | participant | sd reward | 0.05 | | | | |
| random | Residual | sd Observation | 0.13 | | | | |

*Supplementary Table 56:* Full model output for the following linear mixed model: `beta ~ reward + (reward | participant)`, run on the parameter estimates of a drift diffusion model per participant in the congruent condition of experiment 2, where boundary separation $\alpha$, the starting point $\beta$, the drift rate $\delta$, and the non-decision time $\tau$ varied by reward level.

| effect | group | term | estimate | standard error | statistic | df | p-value |
|---|---|---|---|---|---|---|---|
| fixed | | Intercept | 0.51 | 0.01 | 71.30 | 102.00 | p < .001 |
| fixed | | reward | -0.01 | 0.00 | -3.14 | 102.00 | p = .002 |
| random | participant | sd Intercept | 0.06 | | | | |
| random | participant | cor Intercept.reward | -0.18 | | | | |
| random | participant | sd reward | 0.02 | | | | |
| random | Residual | sd Observation | 0.05 | | | | |

*Supplementary Table 57:* Full model output for the following linear mixed model: `delta ~ reward + (reward | participant)`, run on the parameter estimates of a drift diffusion model per participant in the congruent condition of experiment 2, where boundary separation $\alpha$, the starting point $\beta$ and the drift rate $\delta$, and the non-decision time $\tau$ varied by reward level.

| effect | group | term | estimate | standard error | statistic | df | p-value |
|---|---|---|---|---|---|---|---|
| fixed | | Intercept | 0.08 | 0.02 | 4.07 | 102.00 | p < .001 |
| fixed | | reward | -0.02 | 0.01 | -1.85 | 102.00 | p = .068 |
| random | participant | sd Intercept | 0.15 | | | | |
| random | participant | cor Intercept.reward | -0.40 | | | | |
| random | participant | sd reward | 0.06 | | | | |
| random | Residual | sd Observation | 0.17 | | | | |

*Supplementary Table 58:* Full model output for the following linear mixed model: `tau ~ reward + (1 | participant)`, run on the parameter estimates of a drift diffusion model per participant in the congruent condition of experiment 2, where boundary separation $\alpha$, the starting point $\beta$, the drift rate $\delta$, and the non-decision time $\tau$ varied by reward level.

| effect | group | term | estimate | standard error | statistic | df | p-value |
|---|---|---|---|---|---|---|---|
| fixed | | Intercept | 1.00 | 0.02 | 55.68 | 119.10 | p < .001 |
| fixed | | reward | 0.02 | 0.00 | 3.47 | 308.00 | p < .001 |
| random | participant | sd Intercept | 0.17 | | | | |
| random | Residual | sd Observation | 0.07 | | | | |

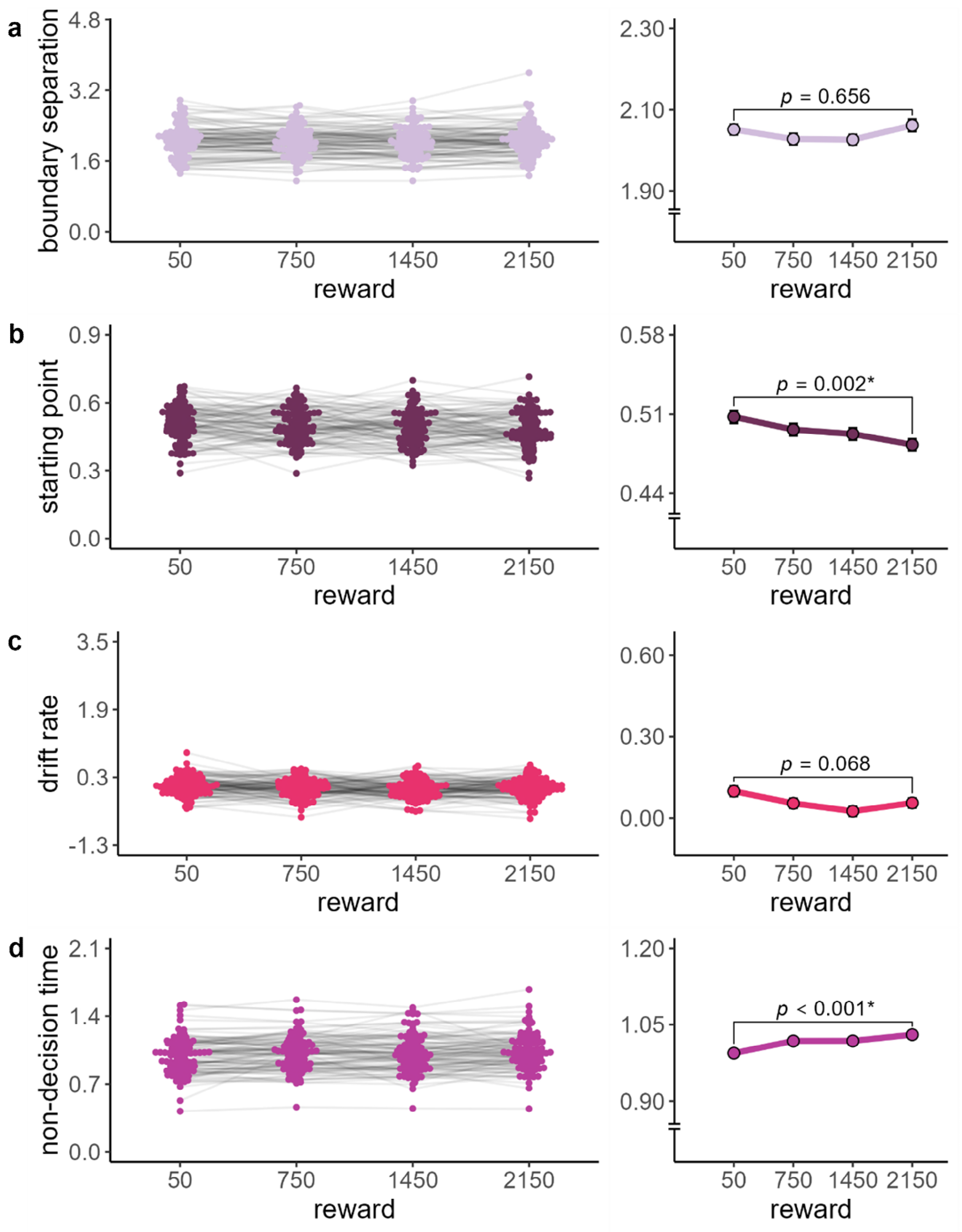


*Supplementary Figure 6:* Parameter estimates for each reward level in the congruent group of experiment 2. Estimates are based on a drift diffusion model run on $n$ = 103 participants with the outcome 'old' (lower boundary) or 'new' (upper boundary), where boundary separation ($\alpha$), the starting point ($\beta$), the drift rate ($\delta$), and the non-decision time ($\tau$) were allowed to vary by reward. **a** $\alpha$ parameter estimates. There was no statistically significant

effect of reward on boundary separation. In the beeswarm plot, light purple dots represent the $\alpha$ estimate per reward level for each participant. Light grey lines connect the dots belonging to the same participant. The line plot shows the mean $\alpha$ estimate for each reward level. The p-value for the reward effect in the mixed model from Supplementary Table 55 is reported. **b** $\beta$ parameter estimates. The starting point decreased as the reward increased. In the beeswarm plot, dark purple dots represent the $\beta$ estimate per reward level for each participant. Light grey lines connect the dots belonging to the same participant. The line plot shows the mean $\beta$ estimate for each reward level. The p-value for the reward effect in the mixed model from Supplementary Table 56 is reported. **c** $\delta$ parameter estimates. Descriptively, the drift rate was lower as the reward increased. In the beeswarm plot, pink dots represent the $\delta$ estimate per reward level for each participant. Light grey lines connect the dots belonging to the same participant. The line plot shows the mean $\delta$ estimate for each reward level. The p-value for the reward effect in the mixed model from Supplementary Table 57 is reported. **d** $\tau$ parameter estimates. The non-decision time increased as the reward increased. In the beeswarm plot, purple dots represent the $\tau$ estimate per reward level for each participant. Light grey lines connect the dots belonging to the same participant. The line plot shows the mean $\tau$ estimate for each reward level. The p-value for the reward effect in the mixed model from Supplementary Table 58 is reported. Black error bars show the within-subject standard error, calculated as implemented in the R package Rmisc[60,61]. Asterisks represent significance at $\alpha = .05$.

### *Incongruent*

*Supplementary Table 59:* Full model output for the following linear mixed model: `alpha ~ reward + (1 | participant)`, run on the parameter estimates of a drift diffusion model per participant in the incongruent condition of experiment 2, where boundary separation $\alpha$, the starting point $\beta$, the drift rate $\delta$, and the non-decision time $\tau$ varied by reward level.

| effect | group | term | estimate | standard error | statistic | df | p-value |
|---|---|---|---|---|---|---|---|
| fixed | | Intercept | 2.07 | 0.03 | 62.43 | 117.01 | p < .001 |
| fixed | | reward | 0.01 | 0.01 | 1.54 | 305.00 | p = .124 |
| random | participant | sd Intercept | 0.32 | | | | |
| random | Residual | sd Observation | 0.13 | | | | |

*Supplementary Table 60:* Full model output for the following linear mixed model: `beta ~ reward + (reward | participant)`, run on the parameter estimates of a drift diffusion model per participant in the incongruent condition of experiment 2, where boundary separation α, the starting point β, the drift rate δ, and the non-decision time τ varied by reward level.

| effect | group | term | estimate | standard error | statistic | df | p-value |
|---|---|---|---|---|---|---|---|
| fixed | | Intercept | 0.52 | 0.01 | 69.25 | 101.00 | p < .001 |
| fixed | | reward | -0.02 | 0.00 | -3.87 | 101.00 | p = .001 |
| random | participant | sd Intercept | 0.06 | | | | |
| random | participant | cor Intercept.reward | -0.51 | | | | |
| random | participant | sd reward | 0.04 | | | | |
| random | Residual | sd Observation | 0.05 | | | | |

*Supplementary Table 61:* Full model output for the following linear mixed model: `delta ~ reward + (reward | participant)`, run on the parameter estimates of a drift diffusion model per participant in the incongruent condition of experiment 2, where boundary separation α, the starting point β, the drift rate δ, and the non-decision time τ varied by reward level.

| effect | group | term | estimate | standard error | statistic | df | p-value |
|---|---|---|---|---|---|---|---|
| fixed | | Intercept | 0.11 | 0.02 | 5.19 | 100.99 | p < .001 |
| fixed | | reward | -0.04 | 0.01 | -3.54 | 100.99 | p < .001 |
| random | participant | sd Intercept | 0.14 | | | | |
| random | participant | cor Intercept.reward | 0.00 | | | | |
| random | participant | sd reward | 0.06 | | | | |
| random | Residual | sd Observation | 0.18 | | | | |

*Supplementary Table 62:* Full model output for the following linear mixed model: `tau ~ reward + (reward | participant)`, run on the parameter estimates of a drift diffusion model per participant in the incongruent condition of experiment 2, where boundary separation $\alpha$, the starting point $\beta$, the drift rate $\delta$, and the non-decision time $\tau$ varied by reward level.

| effect | group | term | estimate | standard error | statistic | df | p-value |
|---|---|---|---|---|---|---|---|
| fixed | | Intercept | 0.98 | 0.02 | 39.54 | 101.00 | p < .001 |
| fixed | | reward | 0.02 | 0.01 | 3.13 | 101.00 | p = .002 |
| random | participant | sd Intercept | 0.24 | | | | |
| random | participant | cor Intercept.reward | -0.02 | | | | |
| random | participant | sd reward | 0.02 | | | | |
| random | Residual | sd Observation | 0.08 | | | | |

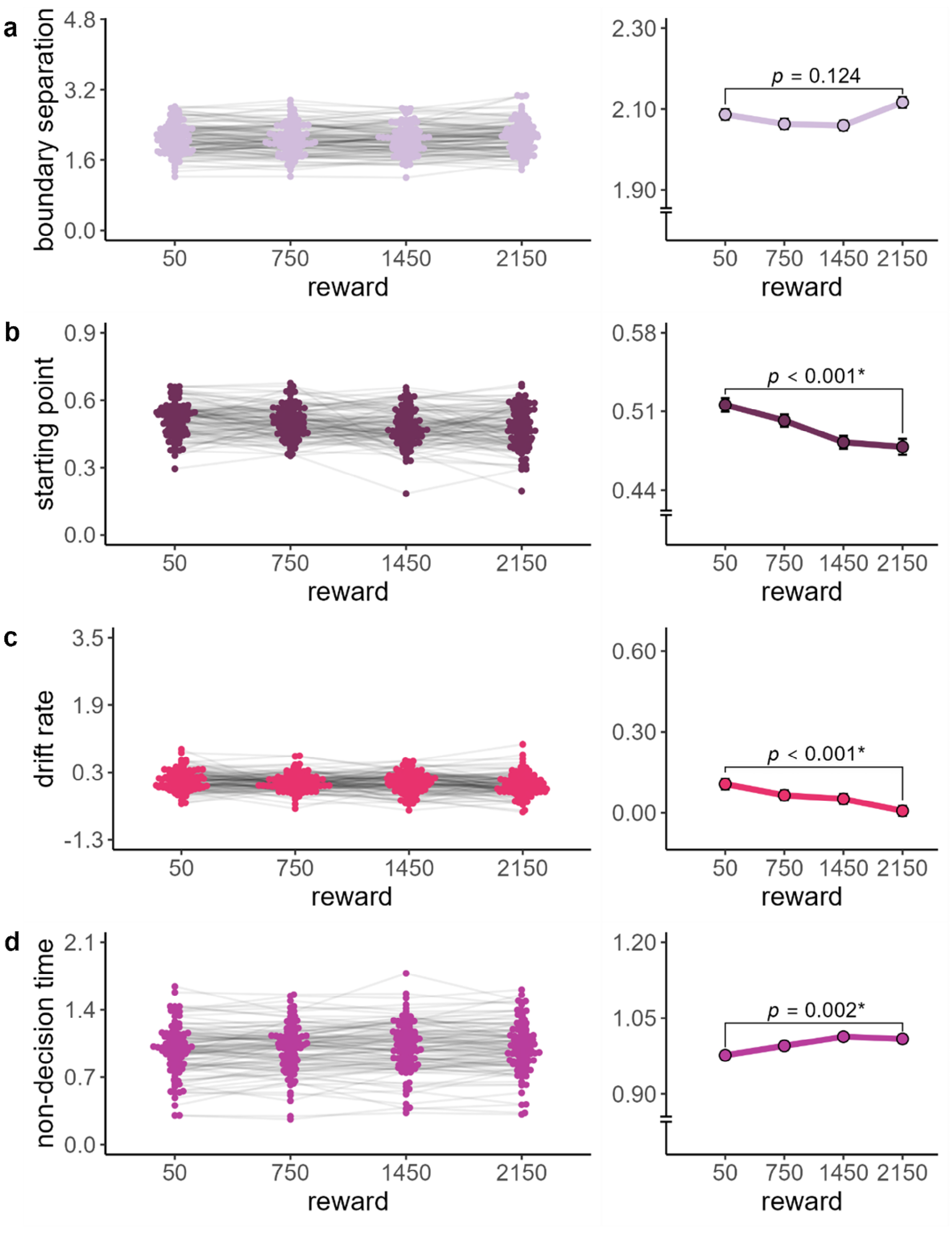


*Supplementary Figure 7:* Parameter estimates for each shown reward level in the incongruent group of experiment 2. Estimates are based on a drift diffusion model run on $n$ = 102 participants with the outcome ‘old’ (lower boundary) or ‘new’ (upper boundary), where boundary separation ($\alpha$), the starting point ($\beta$), the drift rate ($\delta$), and the non-

decision time ($\tau$) were allowed to vary by reward. **a** $\alpha$ parameter estimates. There was no statistically significant effect of reward on boundary separation. In the beeswarm plot, light purple dots represent the $\alpha$ estimate per reward level for each participant. Light grey lines connect the dots belonging to the same participant. The line plot shows the mean $\alpha$ estimate for each reward level. The p-value for the reward effect in the mixed model from Supplementary Table 59 is reported. **b** $\beta$ parameter estimates. The starting point decreased as the reward increased. In the beeswarm plot, dark purple dots represent the $\beta$ estimate per reward level for each participant. Light grey lines connect the dots belonging to the same participant. The line plot shows the mean $\beta$ estimate for each reward level. The p-value for the reward effect in the mixed model from Supplementary Table 60 is reported. **c** $\delta$ parameter estimates. The drift rate decreased as the reward increased. In the beeswarm plot, pink dots represent the $\delta$ estimate per reward level for each participant. Light grey lines connect the dots belonging to the same participant. The line plot shows the mean $\delta$ estimate for each reward level. The p-value for the reward effect in the mixed model from Supplementary Table 61 is reported. **d** $\tau$ parameter estimates. The non-decision time increased as the reward increased. In the beeswarm plot, purple dots represent the $\tau$ estimate per reward level for each participant. Light grey lines connect the dots belonging to the same participant. The line plot shows the mean $\tau$ estimate for each reward level. The p-value for the reward effect in the mixed model from Supplementary Table 62 is reported. Black error bars show the within-subject standard error, calculated as implemented in the R package Rmisc[60,61]. Asterisks represent significance at $\alpha = .05$.

## Experiment 3

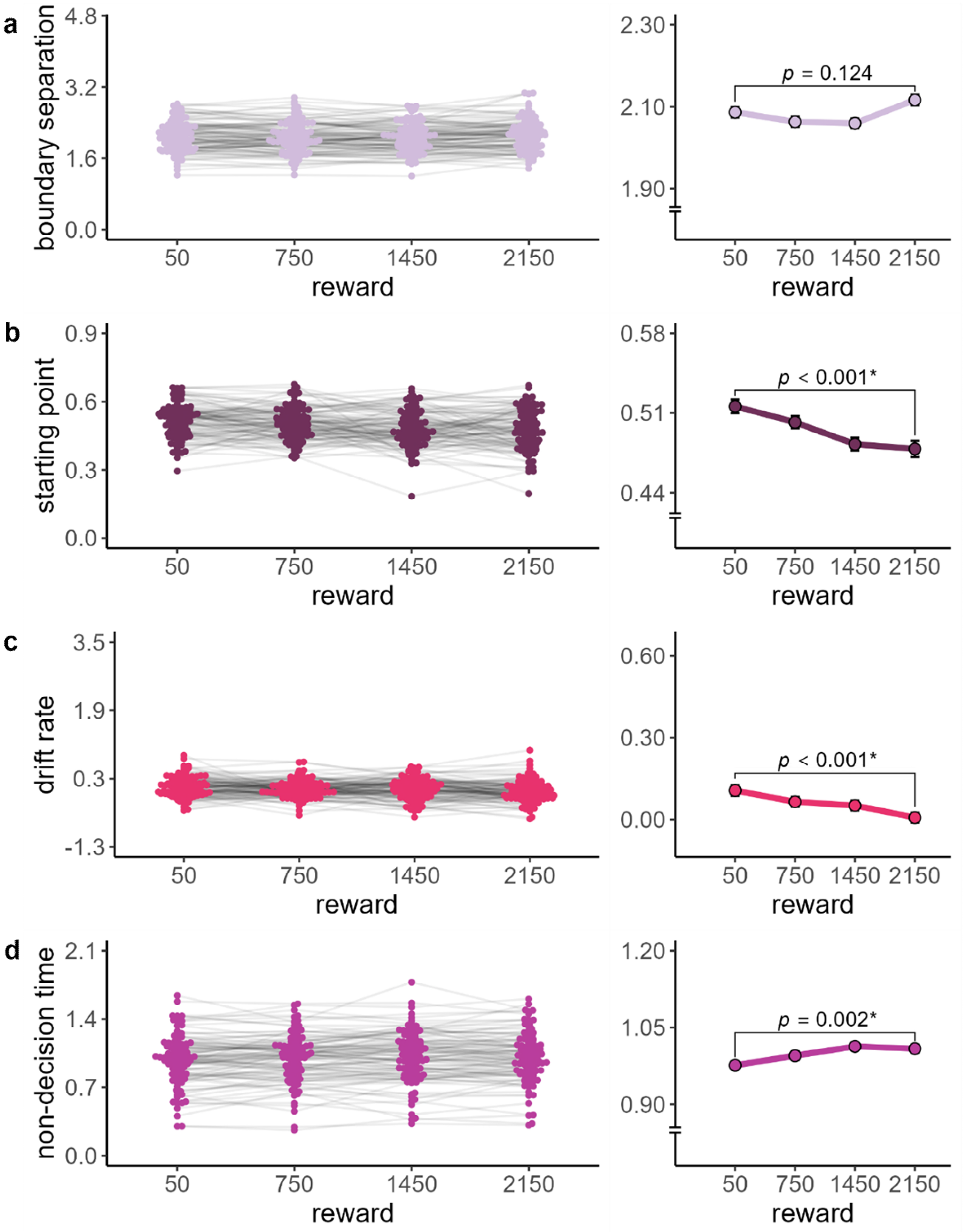


*Supplementary Figure 8:* Parameter estimates for each reward level in experiment 3. Estimates are based on a drift diffusion model run on $N$ = 187 participants with the outcome 'old' (lower boundary) or 'new' (upper boundary), where boundary separation ($\alpha$), the starting point ($\beta$), the drift rate ($\delta$), and the non-decision time ($\tau$) were allowed to

vary by reward. **a** $\alpha$ parameter estimates. There was no significant effect of reward on boundary separation. In the beeswarm plot, light purple dots represent the $\alpha$ estimate per reward level for each participant. Light grey lines connect the dots belonging to the same participant. The line plot shows the mean $\alpha$ estimate for each reward level. **b** $\beta$ parameter estimates. Reward did not affect the starting point. In the beeswarm plot, dark purple dots represent the $\beta$ estimate per reward level for each participant. Light grey lines connect the dots belonging to the same participant. The line plot shows the mean $\beta$ estimate for each reward level. **c** $\delta$ parameter estimates. The drift rate decreased as the reward increased. In the beeswarm plot, pink dots represent the $\delta$ estimate per reward level for each participant. Light grey lines connect the dots belonging to the same participant. The line plot shows the mean $\delta$ estimate for each reward level. **d** $\tau$ parameter estimates. There was no statistically significant effect of reward on non-decision time. In the beeswarm plot, purple dots represent the $\tau$ estimate per reward level for each participant. Light grey lines connect the dots belonging to the same participant. The line plot shows the mean $\tau$ estimate for each reward level. p-values for the reward effect for the $t$-tests reported in the main manuscript (results experiment 3) are shown. Black error bars show the within-subject standard error, calculated as implemented in the R package Rmisc[60,61]. Asterisks represent significance at $\alpha = .05$.

## Supplementary References


1. Hope, R. M. *Rmisc: Ryan Miscellaneous*. (2022).
2. Morey, R. D. Confidence Intervals from Normalized Data: A correction to Cousineau (2005). *Tutor. Quant. Methods Psychol.* **4**, 61–64 (2008).
3. Yonelinas, A. P. & Parks, C. M. Receiver operating characteristics (ROCs) in recognition memory: A review. *Psychol. Bull.* **133**, 800–832 (2007).
4. Bürkner, P.-C. brms: An R Package for Bayesian Multilevel Models Using Stan. *J. Stat. Softw.* **80**, 1–28 (2017).
5. Macmillan, N. A. & Creelman, C. D. *Detection theory: A user's guide, 2nd ed*. xix, 492 (Lawrence Erlbaum Associates Publishers, 2005).
