# Supplementary material for: Memory for rewards guides retrieval
Source: Commun Psychol. 2024 Apr 16;2:31. doi: 10.1038/s44271-024-00074-9 (PMC11332070; doi:10.1038/s44271-024-00074-9)
Supplement: Supplementary file 3 — Reporting Summary [file 44271_2024_74_MOESM3_ESM.pdf]

nature portfolio

Corresponding author(s): juliane.nagel@zi-mannheim.de; gordon.feld@zi.mannheim.de

Last updated by author(s): Mar 5, 2024

Reporting Summary

Nature Portfolio wishes to improve the reproducibility of the work that we publish. This form provides structure for consistency and transparency in reporting. For further information on Nature Portfolio policies, see our Editorial Policies and the Editorial Policy Checklist.

Statistics

For all statistical analyses, confirm that the following items are present in the figure legend, table legend, main text, or Methods section.

n/a	Confirmed	
☐	☒	The exact sample size (*n*) for each experimental group/condition, given as a discrete number and unit of measurement
☐	☒	A statement on whether measurements were taken from distinct samples or whether the same sample was measured repeatedly
☐	☒	The statistical test(s) used AND whether they are one- or two-sided<br>*Only common tests should be described solely by name; describe more complex techniques in the Methods section.*
☐	☒	A description of all covariates tested
☐	☒	A description of any assumptions or corrections, such as tests of normality and adjustment for multiple comparisons
☐	☒	A full description of the statistical parameters including central tendency (e.g. means) or other basic estimates (e.g. regression coefficient) AND variation (e.g. standard deviation) or associated estimates of uncertainty (e.g. confidence intervals)
☐	☒	For null hypothesis testing, the test statistic (e.g. *F*, *t*, *r*) with confidence intervals, effect sizes, degrees of freedom and *P* value noted<br>*Give P values as exact values whenever suitable.*
☐	☒	For Bayesian analysis, information on the choice of priors and Markov chain Monte Carlo settings
☐	☒	For hierarchical and complex designs, identification of the appropriate level for tests and full reporting of outcomes
☐	☒	Estimates of effect sizes (e.g. Cohen's *d*, Pearson's *r*), indicating how they were calculated

Our web collection on statistics for biologists contains articles on many of the points above.

Software and code

Policy information about availability of computer code

Data collection	All tasks were programmed using JsPsych, a collection of JavaScript plugins for browser-based experiments. Participants across all three experiments were recruited via Prolific (https://www.prolific.co/), an online work-sourcing site.
Data analysis	All analyses and plots were generated using R (version 4.3.1 (2023-06-16 ucrt)). Effect sizes were calculated using the R package effectsize (version 0.8.5). (Generalized) linear mixed models were calculated using the R package lme4 (version 1.1.34), and p-values for these models were calculated using the R package lmerTest (version 3.1.3). Drift diffusion models (DDM) were fit using the wdm() function from the R-package RWiener (version 1.3.3). Plots were created using the following packages: ggplot2 (version 3.4.3), ggbeeswarm (version 0.7.2), cowplot (version 1.1.1), ggpubr (version 0.6.0), ggh4x (version 0.2.5), and scales (version 1.2.1). Analysis code has been made available at PsychArchives: https://doi.org/10.23668/psycharchives.13967

For manuscripts utilizing custom algorithms or software that are central to the research but not yet described in published literature, software must be made available to editors and reviewers. We strongly encourage code deposition in a community repository (e.g. GitHub). See the Nature Portfolio guidelines for submitting code & software for further information.

Data

Policy information about availability of data

All manuscripts must include a data availability statement. This statement should provide the following information, where applicable:

- Accession codes, unique identifiers, or web links for publicly available datasets
- A description of any restrictions on data availability
- For clinical datasets or third party data, please ensure that the statement adheres to our policy

All data has been documented and made publicly available at PsychArchives, at the following DOI: https://doi.org/10.23668/psycharchives.13963

Human research participants

Policy information about studies involving human research participants and Sex and Gender in Research.

Reporting on sex and gender	For each study, we recruited an equal number of male and female participants (biological sex) to mirror the distribution of sex in the general population. Participants were asked about their sex and their gender in a demographic questionnaire. For gender, they were able to self-describe a gender label that described their identity best (only between 0 and 3 participants per study/group described themselves as non-binary). We do not report sex- or gender-based analyses in the manuscript because we investigate a general cognitive mechanism (reward-based memory) and do not have any theories why it should differ as function of sex or gender. Before submitting our manuscript, we ensured that sex and/or gender do not influence the main results reported in the manuscript (the effect of reward on hit rate). However, we do not report these analyses, which are beyond the scope of the manuscript. Interested readers can assess the influence of sex/gender based on the data we shared publicly.
Population characteristics	Participants were between 18 and 30 years old, their country of residence was the United Kingdom, their education level was at least A levels (or equivalent) and for the majority (between 73 and 90.2 % per experiment), the mother tongue was English.
Recruitment	Participants across all three experiments were recruited via Prolific (https://www.prolific.co/), an online work-sourcing site. I.e., only participants with access to the internet and enough time to complete online studies (in exchange for receiving compensation) participated in our study.
Ethics oversight	ethics committee of the University Medical Faculty Mannheim, University of Heidelberg

Note that full information on the approval of the study protocol must also be provided in the manuscript.

Field-specific reporting

Please select the one below that is the best fit for your research. If you are not sure, read the appropriate sections before making your selection.

☐ Life sciences ☒ Behavioural & social sciences ☐ Ecological, evolutionary & environmental sciences

For a reference copy of the document with all sections, see nature.com/documents/nr-reporting-summary-flat.pdf

Behavioural & social sciences study design

All studies must disclose on these points even when the disclosure is negative.

Study description	A quantitative study investigating the influence of reward on long-term memory. The effect of reward was investigated in a within-subject design, i.e. the same participants had to memorise different pictures of different reward-values.
Research sample	Participants were between 18 and 30 years old, their country of residence was the United Kingdom, their education level was at least A levels (or equivalent) and for the majority (between 73 and 90.2 % per experiment), the mother tongue was English. They were recruited online via the work-sourcing site Prolific. This sample was chosen for comparability with previous studies (typically young participants with a high education level). An equal number of male and female participants (biological sex) was collected to mirror the distribution in the general population.
Sampling strategy	Participants who were eligible for the study saw the study description on their Prolific homepage and could decide whether they wanted to participate or not. In experiment 1 and 3, the manipulation was within-subjects. For experiment 2, the allocation to the congruent and incongruent group were pseudo-random (equal numbers of participants were collected for each group). For the first experiment, we did not exactly know how many participants were required to detect the effects of interest. We decided to collect N = 200 participants based on our available resources, and because this is a more than 6-fold increase as compared to previous studies. For the following studies, we based our sample size on permutation analyses of the data collected in experiment 1 (see respective preregistrations).

Data collection	Participants completed the study on their computer using their browser. The study was programmed in jsPsych, a collection of JavaScript plugins that facilitate experimental online studies. No researcher supervised the participants directly, i.e. blinding of the researcher was not applicable during data collection. During data analysis, the researchers were not blind to the within- and between-subject labels.
Timing	Experiment 1: 2021-04-26 - 2021-06-22<br>Experiment 2: 2021-08-04 - 2021-09-03<br>Experiment 3: 2022-03-03 - 2022-03-33
Data exclusions	Participants were excluded if they did not complete the study, failed the validation questions twice in a row (ensuring they understood the instructions), their memory was at chance level, they had more than 20% premature responsed or lapses during the Psychomotor Vigilance Task, more than 20% premature responses in the Flanker Task, or responded at chance level during the Flanker Task. All of these exclusion criteria were pre-registered. Additionally, we had to exclude participants with extremely biased reward expectations during experiment 1 (chose the same reward level more than 50% of the time), whose education was below A-levels (desipte giving a different response in the Prolific pre-screening questions), or who participated twice. In experiment 2, two participants with implausibly high performance had to be excluded. For the exact number of participants who were excluded for each reason, please refer to the supplementary material.
Non-participation	For experiment 1 (fina sample: N = 200), 179 participants did not complete both parts of the experiment (of which 109 did not complete the first part of the experiment). For experiment 2, congruent condition, (n = 103) 148 participants did not complete both parts of the experiment (110 of which did not complete the first part of the experiment). For experiment 2, incongruent condition (n = 102), 108 did not complete both parts of the experiment (93 of which did not complete the first part of the study). For experiment 3 (N = 187), 187 did not complete both parts of the experiment (132 of which did not complete the first part of the study). I.e., the majority of participants dropped out early during the experiment.
Randomization	The reward manipulation was within-subjects. For experiment 2, participants were allocated to the congruent or incongruent group pseudo-randomly (equal number of participants in each group).

Reporting for specific materials, systems and methods

We require information from authors about some types of materials, experimental systems and methods used in many studies. Here, indicate whether each material, system or method listed is relevant to your study. If you are not sure if a list item applies to your research, read the appropriate section before selecting a response.

Materials & experimental systems

n/a	Involved in the study
☒	☐ Antibodies
☒	☐ Eukaryotic cell lines
☒	☐ Palaeontology and archaeology
☒	☐ Animals and other organisms
☒	☐ Clinical data
☒	☐ Dual use research of concern

Methods

n/a	Involved in the study
☒	☐ ChIP-seq
☒	☐ Flow cytometry
☒	☐ MRI-based neuroimaging
